# Mature microRNA-binding protein QKI suppresses extracellular microRNA let-7b release

**DOI:** 10.1242/jcs.261575

**Published:** 2024-11-06

**Authors:** Kyung-Won Min, Kyoung-Min Choi, Hyejin Mun, Seungbeom Ko, Ji Won Lee, Cari A. Sagum, Mark T. Bedford, Young-Kook Kim, Joe R. Delaney, Jung-Hyun Cho, Ted M. Dawson, Valina L. Dawson, Waleed Twal, Dong-Chan Kim, Clarisse H. Panganiban, Hainan Lang, Xin Zhou, Seula Shin, Jian Hu, Tilman Heise, Sang-Ho Kwon, Dongsan Kim, Young Hwa Kim, Sung-Ung Kang, Kyungmin Kim, Sydney Lewis, Ahmet Eroglu, Seonghyun Ryu, Dongin Kim, Jeong Ho Chang, Junyang Jung, Je-Hyun Yoon

**Affiliations:** ^1^ Department of Biochemistry and Molecular Biology, Medical University of South Carolina, Charleston, SC 29425, USA; ^2^ Department of Biology, Gangneung-Wonju National University, Gangneung 25457, Republic of Korea; ^3^ Department of Oncology Science, College of Medicine, University of Oklahoma, Oklahoma City, OK 73104, USA; ^4^ Department of Epigenetics and Molecular Carcinogenesis, the University of Texas MD Anderson Cancer Center, Houston, TX 77030, USA; ^5^ Department of Biochemistry, Chonnam National University Medical School, Hwasun 58128, Republic of Korea; ^6^ Neuroregeneration and Stem Cell Programs, Institute for Cell Engineering, Johns Hopkins University School of Medicine, Baltimore, MD 21205, USA; ^7^ Department of Neurology, Johns Hopkins University School of Medicine, Baltimore, MD 21205, USA; ^8^ Regenerative Medicine and Cell Biology, Medical University of South Carolina, Charleston, SC 29425, USA; ^9^ R&D center, NOSQUEST Inc., Seongnam, Gyeonggi 13494, Republic of Korea; ^10^ Department of Pathology and Laboratory Medicine, Medical University of South Carolina, Charleston, SC 29425, USA; ^11^ Department of Cancer Biology, the University of Texas MD Anderson Cancer Center, Houston, TX 77030, USA; ^12^ Department for Pediatric Hematology, Oncology and Stem Cell Transplantation, University Hospital Regensburg, Franz-Josef-Strauss Allee 11, 93053 Regensburg, Germany; ^13^ Department of Cellular Biology and Anatomy, Medical College of Georgia, Augusta University, Augusta, GA 30912, USA; ^14^ Department of Anatomy and Neurobiology, College of Medicine, Kyung Hee University, Seoul 02447, Republic of Korea; ^15^ Department of Pharmaceutical Sciences, College of Pharmacy, University of Oklahoma Health, Sciences Center, Oklahoma City, OK 73117, USA; ^16^ Department of Biology Education, Kyungpook National University, Daegu 41566, Republic of Korea; ^17^ Department of Pathology, College of Medicine, University of Oklahoma, Oklahoma City, OK 73104, USA

**Keywords:** QKI, AGO2, RNA-binding protein, let-7b, Extracellular vesicular miRNA

## Abstract

Argonaute (AGO), a component of RNA-induced silencing complexes (RISCs), is a representative RNA-binding protein (RBP) known to bind with mature microRNAs (miRNAs) and is directly involved in post-transcriptional gene silencing. However, despite the biological significance of miRNAs, the roles of other miRNA-binding proteins (miRBPs) remain unclear in the regulation of miRNA loading, dissociation from RISCs and extracellular release. In this study, we performed protein arrays to profile miRBPs and identify 118 RBPs that directly bind to miRNAs. Among those proteins, the RBP quaking (QKI) inhibits extracellular release of the mature microRNA let-7b by controlling the loading of let-7b into extracellular vesicles via additional miRBPs such as AUF1 (also known as hnRNPD) and hnRNPK. The enhanced extracellular release of let-7b after QKI depletion activates Toll-like receptor 7 (TLR7) and promotes the production of proinflammatory cytokines in recipient cells, leading to brain inflammation in the mouse cortex. Thus, this study reveals the contribution of QKI to the inhibition of brain inflammation via regulation of extracellular let-7b release.

## Teaser

**Summary:** The microRNA-binding protein QKI plays a role in the inhibition of brain inflammation by inhibiting extracellular release of the mature microRNA let-7b and controlling its loading into extracellular vesicles.

## INTRODUCTION

MicroRNAs (miRNAs) assemble into the RNA-induced silencing complex (RISC) with argonaute (AGO) proteins and guide the RISC to target RNAs through base pairing with them (Iwakawa and Tomari, 2022). The miRISC associates with the 3′ untranslated region of a target mRNA, leading to translation repression and degradation (Bagga et al., 2005; Humphreys et al., 2005; Wilczynska and Bushell, 2015). The steady-state levels of miRNAs have been linked to multiple human diseases (Carè et al., 2007; Fiore et al., 2008; Lu et al., 2005), with dysregulation of miRNA particularly in neurological diseases including brain tumors and neurodegeneration (Hébert and De Strooper, 2009; Nicoloso and Calin, 2008). Considering these findings, understanding the molecular mechanisms of how the abundance of miRNAs is regulated could contribute to the prognosis, diagnosis and treatment of diseases.

The cellular concentration of total mature miRNAs was reported to be higher than that of AGO, with experimental limitation in the quantification of mature miRNAs (Janas et al., 2012). We also previously identified that there are plenty of mature miRNAs that are not engaged with AGOs, which was demonstrated by using serial depletion of AGOs, followed by miRNA sequencing and quantification of mature miRNAs from AGO-depleted cells (Min et al., 2024). In addition, numerous studies have shown that miRNAs exist in circulating blood and body fluids (Chen et al., 2008; Mitchell et al., 2008; Weber et al., 2010). These findings indicate that the abundance of cellular miRNAs could be governed by cellular machineries modulating miRNA decay and even excretion of mature miRNAs outside cells.

miRNAs released into extracellular fluids can be found either inside extracellular vesicles (EVs) or bound to AGO2 independently of vesicles (Arroyo et al., 2011; Gallo et al., 2012; O'Brien et al., 2018; Turchinovich et al., 2011; Weaver and Patton, 2020). Mature miRNAs in EVs contain the EXOmotif sequence (CGGAG), which is distinct from cellular retention motifs (CELLmotifs) (Garcia-Martin et al., 2022). Extracellular mature miRNAs are reported to be capable of mediating cell-to-cell communication (Makarova et al., 2021) and are associated with various pathological conditions (Mori et al., 2019). Accumulated studies have demonstrated that miRNAs transported to recipient cells can serve as ligands for Toll-like receptors (TLRs), which are part of the innate immune system that recognizes the invasion of pathogens (Chen et al., 2013; Fabbri et al., 2012; Lehmann et al., 2012). Notably, emerging findings emphasize the roles of TLR7 in sensing extracellular miRNAs in neurological diseases (Buonfiglioli et al., 2019; Lehmann et al., 2012; Yelamanchili et al., 2015). Additionally, extracellular let-7, a mature miRNA, was reported to induce neurodegeneration (Lehmann et al., 2012) and modulate microglial function and glioma growth (Buonfiglioli et al., 2019) by functioning as a ligand of TLR7. Despite the relevance of miRNAs to brain diseases, the regulatory mechanisms of the extracellular miRNA levels are not clearly understood. Notably, a previous study reported that AGO has not been detected in EVs, implicating the existence of novel miRNA-binding proteins (miRBPs) modulating extracellular miRNA release (Jeppesen et al., 2019). Based on these findings, we aimed to profile unknown miRBPs participating in intercellular communication via extracellular miRNAs.

In this study, we showed that several types of RNA-binding proteins (RBPs), including QKI, which is implicated in mRNA splicing, export and decay (De Bruin et al., 2016; Larocque et al., 2002; Thangaraj et al., 2017), and circular RNA biogenesis (Conn et al., 2015), directly bind to a subset of mature miRNAs (let-7, miR-21 and miR-130b). Remarkably, depletion of QKI enhanced extracellular release of let-7b managed by miRBP AU-rich element RBP 1 (AUF1, also known as hnRNPD) and hnRNPK, leading to activation of TLR7 in the recipient cells. Comprehensively, this study reveals a novel role for QKI in brain inflammation and provides novel insights into extracellular miRNA release.

## RESULTS

### QKI is a miRBP

**Fig. 1.**
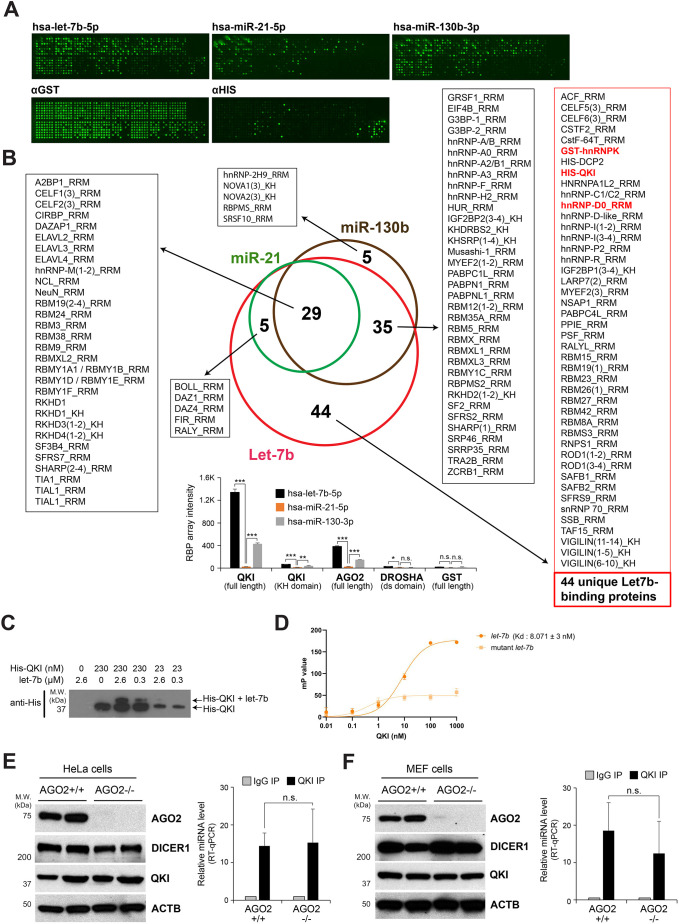
**High-throughput protein array profiles RBPs directly binding with mature miRNAs.** (A) Fluorescence images of biotinylated let-7b, miR-21 and miR-130b bound to proteins spotted on the microarray slides. (B) Venn diagram of RNA-binding proteins (RBPs) commonly identified from the protein array (>500 intensity), followed by lists of RBPs in each overlap. The intensity of let-7b, miR-21 and miR-130b were plotted using values from full-length QKI, its KH domain, full-length AGO2, the double-stranded RNA-binding (ds) domain of DROSHA and full-length GST as a negative control. Bars show mean±s.d. *n*=2. n.s., not significant; *P<0.05, **P<0.01; ***P<0.001 (one-tailed unpaired Student's *t*-test). (C) Western blot analysis of recombinant 6× histidine-tagged QKI alone or in complex with let-7b. His–QKI (23 nM or 230 nM) was incubated with let-7b at room temperature for 1 h, cross-linked with ultraviolet light (254 nm) at 150 mJ/cm^2^, and separated via SDS-PAGE for western blot analysis. The presence of the QKI and let-7b complex was detected by a change of electrophoretic mobility in the blot. The image is representative of three independent experiments. (D) Anisotropy analysis of recombinant QKI protein with let-7b–Cy3 (K_d_=8.071±3 nM) and mutant let-7b lacking the QKI-binding sequence. Bars show mean±s.d. (E,F) Left: western blot analysis of AGO2, DICER1, QKI and ACTB using cell lysates from wild-type and AGO2 knockout (KO) HeLa cells as well as mouse embryonic fibroblasts (MEFs). The images are representative of three independent experiments. Right: ribonucleoprotein (RNP) immunoprecipitation quantitative PCR (RIP-qPCR) analysis of let-7b normalized with *U6* RNA from immunopellets of IgG or anti-QKI antibody using lysates of wild-type or AGO2 KO HeLa cells as well as MEFs. Bars show mean±s.d. *n*=3. n.s., not significant (one-tailed unpaired Student's *t*-test).

The previous low-throughput approach for identifying RBPs interacting with mature miRNAs was limited to the one-by-one examination of RBPs and miRNAs (Zealy et al., 2017). In the present study, we advanced the previous approach for identifying miRBPs by using human protein arrays immobilized with 354 RNA-binding domains (Chen et al., 2020) or ∼17,000 recombinant proteins (Jeong et al., 2012). To identify human proteins interacting with mature miRNAs, we incubated the array slide with the tumor-suppressive miRNA let-7b (Johnson et al., 2005; Lee and Dutta, 2007; Takamizawa et al., 2004), the oncogenic miRNA miR-21 (Asangani et al., 2008; Chan et al., 2005; Meng et al., 2007; Seike et al., 2009) and miR-130b, an miRNA induced during differentiation of adipocytes and a negative regulator of adipogenesis (Lee et al., 2011; Luo et al., 2022; Wang et al., 2013). These miRNAs were labeled at the 5′ end with biotin (Fig. 1A) or internally labeled with Cy5 or DY647 (Jo et al., 2015; Yoon et al., 2015).

In the RNA-binding domain array, we identified 113 RBPs directly interacting with let-7b, 34 interacting with miR-21 and 69 with miR-130b (cutoff <500 original intensity). There were 29 RBPs commonly interacting with three miRNAs, whereas 44 RBPs uniquely bound exclusively with let-7b (Fig. 1B; Table S1). When we profiled RBPs differentially interacting with let-7 family miRNAs (let-7a, let-7b, let-7c and let-7d), we observed that 46 RBPs bound all four miRNAs and 42 bound only let-7b, let-7c and let-7d (Fig. S1A,B).

In the array with ∼17,000 proteins, we identified 60 human proteins that directly interacted with let-7b, 31 that directly interacted with miR-21 and 40 that directly interacted with miR-130b (as normalized to the intensity of GST binding, which was also used for quality control of individual protein spots). Among these 60 proteins, only six interacted with all three miRNAs: (1) ADP-ribosylation factor-like protein 10 (ARL10), (2) splicing factor homolog ISY1, (3) the RBP quaking (QKI), (4) Zic family member 3 (ZIC3), (5) zinc finger protein 490 (ZNF490) and (6) zinc finger protein 597 (ZNF597) (Fig. S1C, Table S2). We did not detect AGO family proteins and let-7b binders, such as HuR (also known as ELAVL1) and AUF1, possibly because of low levels of these proteins on the array (Yoon et al., 2013, 2015) and steric hindrance associated with immobilization of these proteins on the array.

Gene Ontology analysis of the 60 miRNA-interacting proteins revealed that they are involved in diverse cellular processes, such as cation transmembrane transport for let-7b, metal ion binding for miR-21 and DNA-binding for miR-130b (Table S3). Pulldown of mature miRNAs (let-7b, let-7i, miR-141c or miR-200) followed by mass spectrometry also profiled a series of canonical and non-canonical RBPs (Fig. S2A–D) with common protein–protein interaction networks (Fig. S2E). Overall, we successfully identified a number of potential novel miRBPs that interact with let-7b, miR-21 and miR-130b.

QKI is reported as an RBP implicated in mRNA splicing, export and decay (de Bruin et al., 2016; Larocque et al., 2002; Thangaraj et al., 2017), as well as in circular RNA biogenesis (Conn et al., 2015). Notably, photoactivatable ribonucleoside-enhanced crosslinking and immunoprecipitation (PAR-CLIP) analysis showed that mature miRNAs, including let-7b and miR-21, are associated with QKI (Hafner et al., 2010). In addition, we recently reported that QKI functions in miRNA-mediated gene silencing by promoting interaction of AGO2 with let-7b (Min et al., 2024). Considering these findings, we focused on QKI as the RBP that binds with mature miRNAs directly. To verify the protein array results, we performed binding assays of QKI with let-7b, our primary focus in this study, using mobility shift. We incubated recombinant histidine-tagged QKI (purified from *Escherichia coli*) with let-7b, followed by ultraviolet light (254 nm)-induced cross-linking, resolution using SDS-PAGE, and immunoblotting against the 6× histidine tag. Our western blot analysis confirmed that cross-linking of QKI with let-7b resulted in a significantly decreased electrophoretic mobility, suggesting an interaction between QKI and let-7b (Fig. 1C). Our fluorescence polarization assay revealed the binding affinities of recombinant QKI with let-7b (K_d_=8.071±3 nM), miR-21 (K_d_=48.45±17 nM) and miR-130b (K_d_=7.032±3 nM) (Fig. 1D; Fig. S1D). YUAAY-mutant let-7b, lacking the sequence for QKI interaction, failed to bind recombinant QKI, as calculated for mutant let-7b compared to wild-type let-7b (K_d_=8.071±3 nM) (Fig. 1D). This binding affinity is comparable to the high affinity of AGO2 for miRNAs (Tan et al., 2009), suggesting that QKI is a novel miRBP. Ribonucleoprotein (RNP) immunoprecipitation quantitative PCR (RIP-qPCR) of QKI enriched let-7b in HeLa cells and mouse embryonic fibroblasts (MEFs), regardless of the presence of AGO2 (Fig. 1E,F).

The QKI-binding sites on target mRNAs have previously been characterized with the conserved motif YUAAY (Teplova et al., 2013) and ACUAAY(N1–20)UAAY using systematic evolution of ligands by exponential enrichment (SELEX) (Galarneau and Richard, 2005), and AYUAAY using PAR-CLIP (Hafner et al., 2010). Although none of these motifs are present in let-7b (5ʹ-UGAGGUAGUAGGUUGUGUGGUU-3ʹ), miR-21 (5ʹ-UAGCUUAUCAGACUGAUGUUGA-3ʹ) or miR-130b (5ʹ-CAGUGCAAUGAUGAAAGGGCAU-3ʹ), the top 20 miRNAs identified from QKI PAR-CLIP share the motif WSUDSU (identified by the MEME suite) (Bailey et al., 2009), which is different from the aforementioned three motifs.

To support this finding, we performed simulated molecular docking of QKI with let-7b, miR-21 and miR-130b. Although the overall bound conformation of let-7b was not a perfect fit for that of the substrate (Fig. S3A,B), the UAA sequence in the YUAAY motif fit well with the UGU sequence in the let-7b RNA (Fig. S3C). However, our binding affinity calculations showed that there are no critical motifs in let-7b for binding QKI (Fig. S3D–F). These data indicate that QKI can bind let-7b, which does not have any consensus sequence (Galarneau and Richard, 2005; Hafner et al., 2010; Teplova et al., 2013). The fitting of both miR-21 and miR-130b was similar to that of let-7b, with no essential motifs for binding to QKI (Fig. S3G–L).

### QKI depletion promotes extracellular release of let-7b

**Fig. 2.**
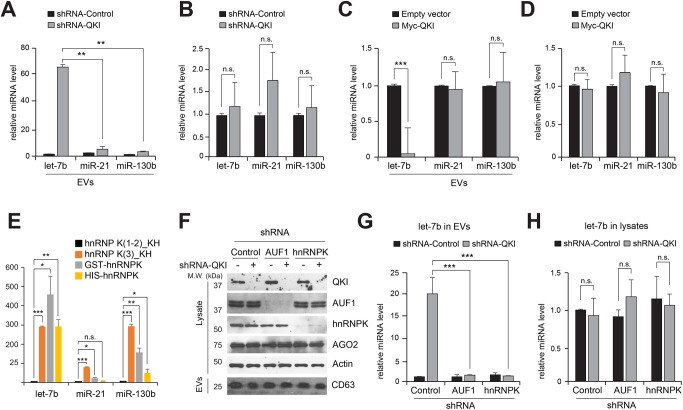
**QKI suppresses extracellular release of let-7b.** (A,B) RT-qPCR analysis of let-7b, miR-21 and miR-130b in (A) extracellular vesicles (EVs) and (B) total RNA from HeLa cells transfected with control or QKI shRNA for 48 h. *n*=3. n.s., not significant; **P<0.01 (one-tailed unpaired Student's *t*-test). (C,D) RT-qPCR analysis of let-7b, miR-21 and miR-130b in (C) EVs and (D) total RNA from HeLa cells transfected with empty vector or Myc–QKI plasmids for 48 h. *n*=3. n.s., not significant; **P<0.01 (one-tailed unpaired Student's *t*-test). (E) Mature miRNA intensity from RNA-binding domain arrays containing hnRNPK full-length proteins and KH domains. *n*=2. n.s., not significant; *P<0.05; **P<0.01; ***P<0.001 (one-tailed unpaired Student's *t*-test). (F) Immunoblot analysis of CD63 (from EVs) and QKI, AUF1, hnRNPK, AGO2 and actin (from cell lysates) in HeLa cells transfected with shRNAs for 48 h. The images are representative of three independent experiments. (G,H) RT-qPCR analysis of let-7b in (G) EVs and (H) total RNA from HeLa cells transfected with shRNAs for 48 h. *n*=3. n.s., not significant; ***P<0.001 (one-tailed unpaired Student's *t*-test). Bars in A–E,G,H show mean±s.d.

The existence of other miRBPs, in addition to AGO proteins, prompted us to speculate that QKI titrates the availability of AGO2-free miRNAs for packaging into EVs for paracrine signaling. Thus, we hypothesized that QKI regulates EV-mediated miRNA release. To test this, EVs were purified using sucrose density gradient centrifugation as performed previously (Kang et al., 2024; Kwon et al., 2016) (Fig. S4A,B). We first compared the levels of QKI target miRNAs (let-7b, miR-21 and miR-130b) in EVs from QKI shRNA-treated and control cells. Reverse transcription quantitative PCR (RT-qPCR) analysis showed that the level of let-7b in EVs was significantly higher in QKI shRNA-treated cells than in control cells (Fig. 2A); the levels of miR-21 and miR-130b in EVs were not increased significantly in QKI shRNA-treated cells compared to control cells. We also compared the expression of these three miRNAs inside cells and found that the levels of cellular miRNAs did not change significantly in QKI shRNA-treated cells (Fig. 2B). Small RNA-sequencing analysis demonstrated that, among let-7 family miRNAs, let-7b and let-7c were enriched in EVs after silencing QKI in HeLa cells (Fig. S4A). The abundance of let-7b from whole medium or mouse serum before EV purification exhibited similar changes to those after EV purification (Fig. S4C). In contrast, overexpression of QKI significantly decreased the abundance of let-7b in EVs compared to that in empty vector control-treated cells (Fig. 2C), with no significant difference in cytosolic miRNA expression (Fig. 2D) These results demonstrate that QKI prevents release of let-7b via EVs.

Next, we reasoned that if QKI depletion promotes let-7b accumulation in EVs, there should be cargo proteins to carry the let-7b out of cells. To address this question, we used mass spectrometry to profile proteins loaded into EVs. Among 321 proteins, we identified five RBPs (AUF1, DHX9, eIF4A1, hnRNPH and hnRNPK), but not QKI, AGO2 or HuR. The absence of AGO2 from our EV protein profiling is consistent with the recent finding in a previous publication (Jeppesen et al., 2019). RNA-binding domain array data also identified a KH domain critical for miRNA binding (Fig. 2E).

Among the five RBPs, AUF1 and hnRNPK are known to interact directly with let-7b and miR-21 (Ray et al., 2013; Yoon et al., 2015). Depletion of AUF1 or hnRNPK, in combination with QKI shRNA (Fig. 2F) suppressed let-7b enrichment in EVs (Fig. 2G), although the overall level of let-7b was unchanged (Fig. 2H). When we compared the levels of the exosomal protein CD63, its abundance did not change significantly (Fig. 2F). Our findings demonstrate that the let-7b-binding proteins AUF1 and hnRNPK are responsible for accelerated let-7b release when QKI is silenced. In this study, as we did not scrutinize the intracellular events underpinning the engagement of AUF1 and hnRNPK in the miRNA-packing process for exosomal release, it is plausible that these proteins could be involved in the LC3-conjugation machinery for specifying the cargo packages into EVs (Leidal et al., 2020). This suggests that increase of the AUF1/hnRNPK-let-7b complex when QKI levels are limited could contribute to let-7b release.

### QKI deficiency promotes inflammation of neuronal cells

A subset of extracellular miRNAs has been reported as ligands for the TLR family sensing viral single-stranded RNAs, which in turn prompted us to hypothesize that EV-loaded let-7b activates the TLR7 signal in endosomes of recipient cells to promote inflammatory responses in the central nervous system (CNS)/brain and cancer cells (Buonfiglioli et al., 2019; Fabbri et al., 2012; Lehmann et al., 2012). This led us to examine whether accelerated exosomal release of let-7b, upon QKI depletion, activates TLR7 and subsequent inflammation.

**Fig. 3.**
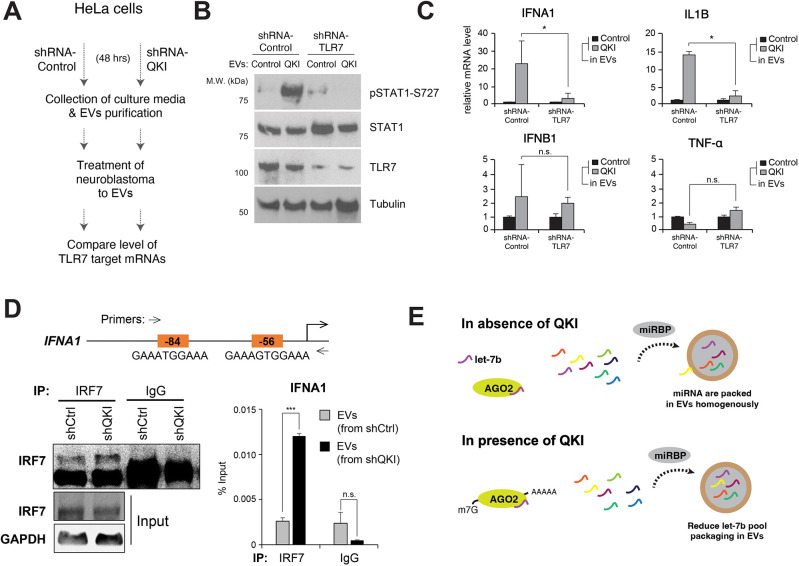
**QKI depletion activates TLR7 signaling.** (A) Experimental procedure for treating the recipient cells (SH-SY5Y) with extracellular vesicles (EVs) purified from HeLa cells transfected with control or QKI shRNA. (B) Immunoblot analysis of phosphorylated (p)STAT1-S727, STAT1, TLR7 and tubulin in SH-SY5Y cells treated with EVs derived from control or QKI shRNA-transfected HeLa cells for 2 h. The images are representative of three independent experiments. (C) RT-qPCR analysis of *IFNA1*, *IL1B*, *IFNB1* and *TNFA* mRNA levels in TLR7 shRNA-transfected SH-SY5Y cells treated with EVs derived from control or QKI shRNA-transfected HeLa cells for 2 h. Bars show mean±s.d. *n*=3. n.s., not significant; *P<0.05 (one-tailed unpaired Student's *t*-test). (D) Chromatin immunoprecipitation-quantitative PCR (ChIP-qPCR) analysis of IRF7 occupancy on its target gene *IFNA1* in the EV-stimulated recipient cells. Schematic diagrams show canonical IRF7 binding sites on *IFNA1* promoter region, and PCR amplicons indicated by arrows. The bar graphs show relative changes in the level of IRF7 binding on *IFNA1* promoter regions normalized to input chromatin. Bars show mean±s.d. *n*=3. n.s., not significant; ***P<0.001 (one-tailed unpaired Student's *t*-test). (E) Schematic diagram of the proposed function of QKI in miRNA metabolism. miRBP, miRNA-binding protein.

To this end, we compared the abundance of four TLR7-target mRNAs [*IFNA1*, *IFNB1*, *IL1B* and *TNFA* (also known as *TNF*)] in the recipient cells (SH-SY5Y), after the cells were treated with EVs isolated from control or QKI shRNA-treated cells (HeLa) (Fig. 3A). Our RT-qPCR analysis showed that the recipient cells exposed to EVs from QKI shRNA-treated cells significantly increased the phosphorylation of STAT1 at Ser727 in a TLR7-dependent manner (indicating TLR7 activation) (Fig. 3B) (Ramirez-Ortiz et al., 2015).

We also observed that neuroblastoma cells exposed to EVs from QKI-depleted HeLa cells expressed significantly more *IFNA1* and *IL1B* mRNA than did neuroblastoma cells exposed to EVs from control shRNA-treated cells. However, the abundance of *IFNB1* and *TNFA* mRNA was not changed significantly (Fig. 3C). More strikingly, induction of *IFNA1* and *IL1B* mRNAs was suppressed by depletion of TLR7 in the recipient cells (Fig. 3C). TLR7 activation was also confirmed by measuring IRF7 occupancy on the promoter regions of *IFNA1* (Fig. 3D). Our results show that EVs originating from QKI-depleted donor cells significantly increase the degree of IRF7 binding to its target gene promoters in comparison with EVs from control shRNA-treated cells. Our findings above demonstrate that accelerated let-7b release via EVs after QKI depletion activates the TLR7 pathway in the recipient cells (Fig. 3E).

### Qki deficiency in mice promotes activation of microglia surrounding TLR7-overexpressing neurons

If QKI prevents extracellular let-7b release and subsequent inflammatory responses, Qki-expressing cells in mice could function in sequestering let-7b inside cells. To determine which cell types express Qki (Qki5, Qki6, Qki7 and Qki7b isoforms) in the cerebral cortex, we performed immunostaining with antibodies detecting proteins expressed exclusively in one cell type of mouse brain: NeuN (encoded by *Rbfox3*) or NF (encoded by *Nefm*) for neurons, GFAP for astrocytes, CNPase (encoded by *Cnp*) for oligodendrocytes, and Iba1 (encoded by *Aif1*) for microglia, respectively. We also used antibodies recognizing Qki5 only (anti-Qki5 antibody) or all Qki isoforms (anti-pan-Qki antibody) (see more details in the Materials and Methods).

**Fig. 4.**
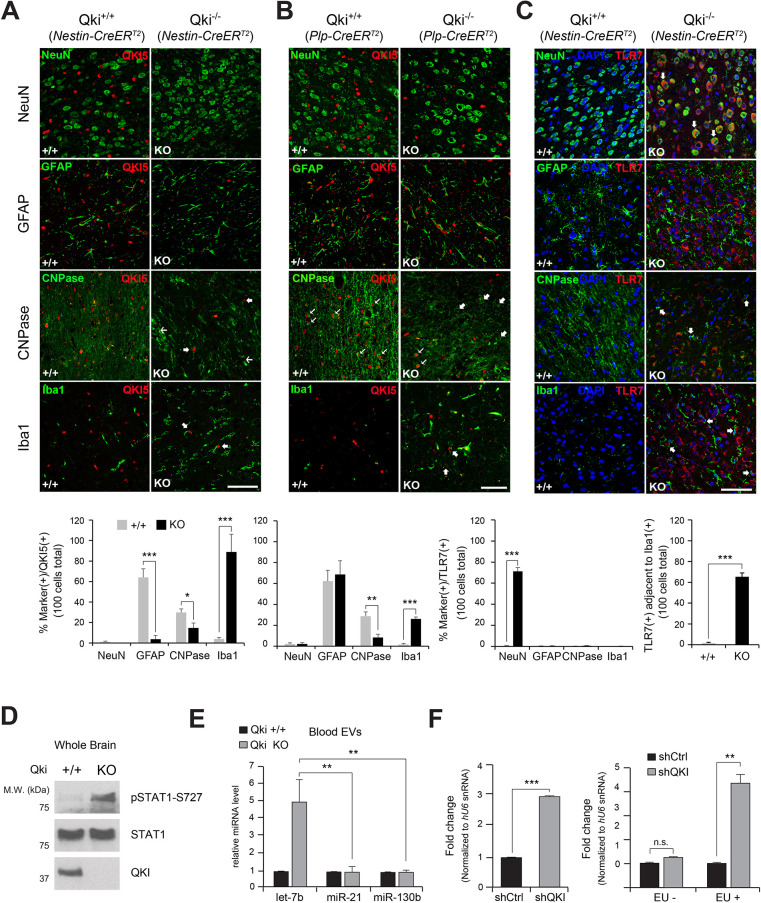
**Expression and function of Qki in neurons, astrocytes, microglia and oligodendrocytes of mouse cerebral cortex.** (A,B) Top: immunolabeling of Qki5 (red) with NeuN, GFAP, CNPase and Iba1 (green) using sections of *Nestin-CreER^T2^* (A) and *Plp-CreER^T2^* (B) *Qki^+/+^* and *Qki* KO mice. In CNPase-stained images, thick and thin arrows indicate Qki5-positive and -deficient oligodendrocytes. In Iba1-stained images, thick arrows indicate Qki5-positive microglia. Scale bars: 50 μm. Bottom: marker/Qki5 double-positive cells were counted with cell marker immunolabeling out of 100 Qki5-positive cells. The number of marker-stained cells out of 100 Qki5-positive cells compared with that of the control. Bars shown mean±s.d. *n*=3. *P<0.05; **P<0.01; ***P<0.001 (one-tailed unpaired Student's *t*-test). (C) Top: confocal images of immunolabeling using sections from *Nestin-CreER^T2^ Qki^+/+^* and *Qki* KO mice with TLR7 (red) with NeuN, GFAP, CNPase, and Iba1 (green). Scale bar: 50 μm. DAPI is a marker of nucleus. In NeuN-stained images, arrows indicate NeuN/TLR7 double-positive neurons. In CNPase-stained images, arrows indicate TLR7-negative oligodendrocytes. In Iba1-stained images, arrows indicate microglia wrapping around TLR7-positive neurons. TLR7-positive cells were counted with TLR7 immunolabeling out of 100 DAPI-positive cells in *Nestin-CreER^T2^ Qki* samples. Quantitative data indicate counts of TLR7-positive cells surrounded by Iba1-positive cells among 100 DAPI-positive cells in *Nestin-CreER^T2^ Qki* samples as the inflammatory index. Bars shown mean±s.d. *n*=3. ***P<0.001 (one-tailed unpaired Student's *t*-test). (D) Western blot analysis of pSTAT1-S727 and Qki in brain sections from *Qki^+/+^* and *Qki^−/−^* mice. Images are representative of three independent sections. (E) RT-qPCR analysis of let-7b, miR-21 and miR-130b in blood extracellular vesicles (EVs) from *Qki^+/+^* and *Qki* KO mice. Bars shown mean±s.d. *n*=3. **P<0.01 (one-tailed unpaired Student's *t*-test).

First, we analyzed the expression patterns of Qki5 in *Nestin-CreER^T2^ Qki^+/+^* and *Nestin-CreER^T2^ Qki^−/−^* mice, which have Qki deleted (exon 2 common in all isoforms; Shingu et al., 2017) (Fig. 4A,B) in neuroectoderm-originated cells, such as neurons, astrocytes and oligodendrocytes. Qki5 staining was mainly observed in nuclei, which overlapped with GFAP, CNPase and Iba1 but not with NeuN signals in *Qki^+/+^* mice (Fig. 4A). In the cerebral cortex from *Plp-CreER^T2^ Qki^+/+^* mice, the Qki5 signal overlapped with signals from GFAP and CNPase as well (Fig. 4B). Strikingly, the *Nestin-CreER^T2^ Qki^−/−^* genotype resulted in the depletion of Qki5 in astrocytes and oligodendrocytes, whereas the *Plp-CreER^T2^ Qki^−/−^* genotype resulted in the depletion of Qki5 only in oligodendrocytes (Fig. 4A,B). Residual staining from anti-Qki5 and anti-pan-Qki antibodies in *Qki^−/−^* mice might come from Qki in microglia or truncated Qki proteins containing at least exon 1, considering *Qki^−/−^* as a functional knockout (KO).

In quantitative analyses of Qki5-positive cells, *Nestin-CreER^T2^ Qki* KO showed the knockout from 64±8.3% to 3.7±3.7% in astrocytes and from 29.8±3.4% to 14.8±4.7% in oligodendrocytes (Fig. 4A, bottom). *Plp-CreER^T2^ Qki* KO showed the knockout from 62.2±10.4% to 68.7±13% in astrocytes and from 28.7±4.1% to 8.3±3.1% in oligodendrocytes (Fig. 4B, bottom). Additionally, both *Nestin-CreER^T2^* and *Plp-CreER^T2^ Qki* KO showed no alteration of Qki5 expression in microglia compared with that in each control, as microglia originate from the mesoderm but not the neuroectoderm, which are affected by the *Nestin* or *Plp* promoter, respectively. Intriguingly, both *Nestin-CreER^T2^* and *Plp-CreER^T2^ Qki* KO showed increased Iba1 expression from 100 stained Qki5-positive cells, indicating increase of neuroinflammation. The structural abnormality of oligodendrocytes in *Qki^−/−^* mice also supports our findings. Thus, these results demonstrate that Qki is expressed in astrocytes and oligodendrocytes of the normal cerebral cortex and its knockout was accomplished successfully for inducing microglial activation in both *Nestin-CreER^T2^* and *Plp-CreER^T2^ Qki* KO mice.

To determine how Qki deficiency in the cerebral cortex promotes brain inflammation via let-7b, we traced cell types targeted by let-7b in Qki-deficient brains. As let-7b is recognized by its receptor, TLR7, we reasoned that TLR7-expressing cells are mainly affected by let-7b, the release of which is promoted by Qki deficiency. To address this, we performed immunostaining using sections from *Nestin-CreER^T2^ Qki^+/+^* and *Qki* KO mice. Qki deletion increased TLR7 signals, which exclusively overlapped with NeuN signals (Fig. 4C) but not pan-Qki (Fig. S4E,F) expressed in microglia (Fig. S4G,H). Staining of double-stranded RNAs using the J2 antibody also mainly overlapped with NF only in *Qki* KO samples (Fig. S5A), without significant changes in staining of their receptors, TLR3 (Fig. S5B). Staining of J2 and TLR7 also overlapped only in *Qki* KO samples (Fig. S5D) implicating overlapping function of let-7b and double-stranded RNAs. Strikingly, microglia stained with Iba1 surrounded neurons stained with NeuN and TLR7 in *Nestin-CreER^T2^ Qki^−/−^* compared with *Qki^+/+^* samples (Fig. 4C). These results suggest that neuroinflammation induced by Qki deletion is initiated independent of microglia. Altogether, Qki deletion mainly happens in oligodendrocytes, resulting in destruction of their process structure and recruitment of microglia to neurons wrapped abnormally by processes of Qki-deficient oligodendrocytes.

**Fig. 5.**
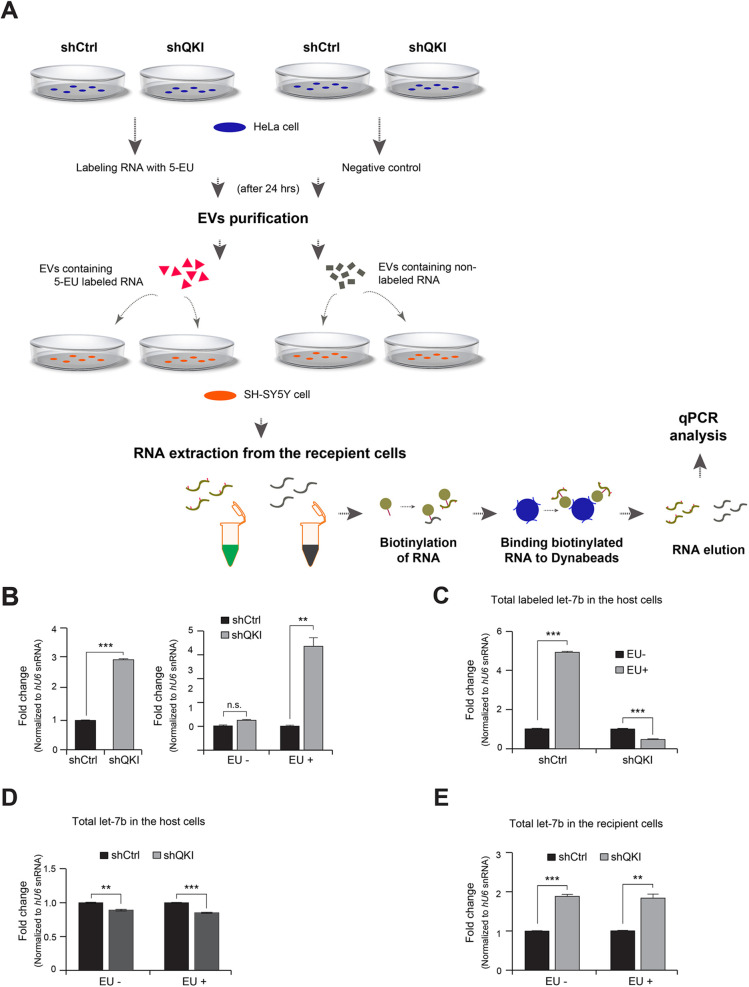
**Tracking of let-7b transfer from host HeLa to recipient SH-SY5Y cells.** (A) Schematic diagram of the experimental design for tracking the labeled let-7b originating from QKI-deficient or control donor cells to the recipient cells via extracellular vesicles (EVs). Donor cells were grown in the presence of 5-EU to label newly synthesized RNA transcripts. EVs from the donor cells were isolated and applied to the recipient cells grown under standard conditions without 5-EU labeling. After 6 h, EV-treated or untreated cells were lysed and the presence of labeled or unlabeled let-7b was determined by RT-qPCR. (B) Tracking of labeled let-7b derived from control or QKI shRNA-transfected cells (HeLa) to recipient cells (SH-SY5Y). Total RNA was extracted from the EV-treated recipient cells; then, 5-EU-labeled RNA in the pool of total RNA was precipitated and biotinylated. The presence of labeled let-7b was determined by qPCR normalized with *U6* snRNA. *n*=3. n.s., not significant; **P<0.01; ***P<0.001 (one-tailed unpaired Student's *t*-test). (C,D) Analysis of total let-7b in the host HeLa cells. *n*=2. **P<0.01; ***P<0.001 (one-tailed unpaired Student's *t*-test). (E) Analysis of total let-7b in the recipient SH-SY5Y cells stimulated with EVs purified from either control or QKI-depleted HeLa cells. *n*=2. **P<0.01; ***P<0.001 (one-tailed unpaired Student's *t*-test). Bars in B–E show±s.d.

To examine increase in inflammation in Qki-deficient brains, we studied sections of brains from *Qki^+/+^* and *Qki^−/−^* mice (Shingu et al., 2017). Brain lysates from *Qki^−/−^* mice exhibited significantly increased levels of phosphorylated Stat1 at Ser727 (Fig. 4D). Similar to our observations in EVs from QKI-depleted human cells, blood EVs purified from *Qki^−/−^* mouse sera were enriched in the miRNA let-7b, but not miR-21 and miR-130b (Fig. 4E). The abundance of let-7b in the whole sera of *Qki^−/−^* mice also showed similar changes compared to that in *Qki^+/+^* mice (Fig. S4C). The let-7b released from the host cells was indeed absorbed into the recipient cells, based on our results from miRNA labeling with ethynyl-labeled uridine (5-EU) and quantification in the host and recipient cells with RT-qPCR (Fig. 5). Our results support our hypothesis that let-7b in EVs acts as a paracrine signal. These results confirm that QKI is required for suppression of CNS/brain inflammation via regulation of the let-7b–TLR7 pathway.

## DISCUSSION

It has been reported that the biosynthesis of miRNA, a major regulator of gene expression influencing various aspects within the cell, is tightly regulated by multiple RBPs (Rüegger and Großhans, 2012; Treiber et al., 2017). Recently, a number of studies have reported that RBPs directly interact with mature miRNA (Treiber et al., 2017; Yoon et al., 2014, 2015), indicating that RBPs are involved in the regulation of mature miRNA function as well as in the biosynthesis of miRNA. In this study, we profiled the RBPs associated with miRNAs with different functions to identify RBP candidates essential for the function of each miRNA (Fig. 1A,B). Interestingly, we discovered that the most RBPs binding with let-7b are not AGO family proteins (Fig. 1B), despite them being known to mediate miRNA-mediated gene silencing (Fabian and Sonenberg, 2012; Behm-Ansmant et al., 2006; Schmitter et al., 2006). These results imply that the activity or abundance of let-7b is tightly regulated by multiple RBPs.

Recently, extracellular let-7b was reported to act as a direct ligand of RNA-sensing TLR7 in macrophages and neurons (Lehmann et al., 2012). We hypothesized that the RBP identified by protein arrays might function in the extracellular release of let-7b and demonstrated that QKI-mediated suppression of let-7b release contributes to brain inflammation via regulation of intercellular communication (Fig. 4). In addition, we have shown that depletion of QKI in HeLa cells enhances let-7c abundance in EVs (Fig. S4A). Although let-7c is not a ligand of TLR7, these results indicate that QKI functions in the loading of let-7c as well as let-7b into EVs and suggest that let-7c is also associated with brain inflammation by silencing let-7 target genes in the recipient cells. Previously, we revealed that QKI promotes AGO2/let-7b-mediated gene silencing and functions as a tumor suppressor in HeLa cells (Min et al., 2024). We speculate that the presence of QKI enhances the role of let-7b in gene silencing, whereas the absence of QKI accelerates the loading of let-7b into EVs (Fig. 3E). In conclusion, QKI is a key mediator modulating the function of mature let-7b as a master regulator of brain inflammation (Fig. 3E).

Notably, our results showed that QKI depletion did not affect the abundance of miR-21 and miR-130b in the EVs (Fig. 2), which suggests that the regulatory mechanism of miR-21 and miR-130b loading into EVs could be different from that of let-7b. All in all, we profiled various RBP candidates for mature miRNAs, and our findings can be utilized to elucidate the regulatory mechanisms of miRNA loading into EVs in multiple human diseases. Collectively, our research contributes to our understanding of extracellular miRNA metabolism and provides novel insights into it.

## MATERIALS AND METHODS

### Cell culture, transfection, shRNAs and plasmids

Human HeLa, MEFs and SH-SY5Y cells were cultured in Dulbecco's modified Eagle medium (DMEM; Invitrogen) supplemented with 10% (v/v) fetal bovine serum (FBS; Corning, #35-015-CV) and antibiotics. Using polyethylenimine, the cells were transfected with shRNA plasmids designed to QKI (5′-GCTCAGAACAGAGCAGAAATC-3′) (Novikov et al., 2011), AUF1 (5′-CGAAGGAACAATATCAGCAA-3′), hnRNPK (5′-GATGTTTGATGACCGTCGCG-3′) or TLR7 (5′-CAGGAGTCTGACGAAGTATT-3′). pcDNA3 (Invitrogen) and pDESTmycQKI (Addgene, #19870) were used for overexpression experiments. Cells were collected for RT-qPCR, western blotting and EV purification 48 h after transfection.

### RNP analysis

For immunoprecipitation (IP) of RNP complexes from cell lysates (RIP analysis) (Yoon et al., 2012), cells were lysed in protein extraction buffer (PEB) with 20 mM Tris-HCl at pH 7.5, 100 mM KCl, 5 mM MgCl_2_ and 0.5% NP-40 for 10 min on ice, and centrifuged at 10,000 ***g*** for 15 min at 4°C. The supernatant was then incubated for 1 h at 4°C with protein A-Sepharose beads coated with antibodies recognizing QKI (1 µg/1 ml lysate, ab126742, Abcam), AGO2 (1 µg/1ml lysate, ab57113, Abcam) or control IgG (1 µg/1 ml lysate, sc-2025, Santa Cruz Biotechnology). After the beads were washed with NT2 buffer (50 mM Tris-HCl at pH 7.5, 150 mM NaCl, 1 mM MgCl_2_ and 0.05% NP-40), the complexes were incubated for 15 min at 37°C with 20 units of RNase-free DNase I (EN0521, Thermo Fisher Scientific). They were finally incubated for 15 min at 55°C with 0.1% SDS and 0.5 mg/ml Proteinase K (KB-0111, Bioneer) to remove the remaining DNA and proteins, respectively. The RNA isolated from the IP materials was further assessed by RT-qPCR analysis using the primers given in Table S4. Normalization of RIP results was carried out by quantifying, in parallel, the relative levels of housekeeping RNAs such as *GAPDH* mRNA and *U6* snRNA in each IP sample. These abundant RNAs are non-specifically recovered during IP reactions.

### Western blot analysis

Whole-cell lysates prepared in PEB were separated by SDS-polyacrylamide gel electrophoresis (SDS-PAGE) and transferred onto nitrocellulose membranes (Invitrogen iBlot Stack). Anti-β-actin (sc-47778), anti-IRF7 (sc-74472) and anti-α-tubulin (sc-5286) antibodies were purchased from Santa Cruz Biotechnology. Anti-Dicer (ab82539), anti-AGO2 (ab57113), anti-CD63 (ab59479) and anti-QKI (ab126742) antibodies were purchased from Abcam. Anti-pSTAT1 (8826S), anti-GAPDH (2118S), anti-hnRNPK (9081S) and anti-STAT1 (14994S) antibodies were purchased from Cell Signaling Technology. Anti-TLR7 (NBP2-24906) antibody was purchased from Novus Biologicals. Anti-AUF1 (07-260) antibody was purchased from Millipore. All primary antibodies were used at 1:1000. The HRP-conjugated secondary antibodies (1:5000, 7074S) were purchased from GE Healthcare.

### RNA analysis

For RIP analysis, Ribozole (Amresco) was used to extract total RNA, and acidic phenol (Ambion) was used to extract RNA. Reverse transcription was performed using random hexamers and reverse transcriptase (Maxima, Thermo Fisher Scientific), and real-time quantitative (q)PCR using gene-specific primers (Table S4) and SYBR Green Master Mix (Kapa Biosystems) using a Bio-Rad iCycler instrument.

miRNA quantitation was performed after RNA extraction from immunoprecipitated samples (with anti-AGO2 antibody or control IgG), polyadenylation (QuantiMiR kit, #RA420A, System Biosciences), and hybridization with oligo-dT adaptors. After reverse transcription, cDNAs were quantitated by qPCR with miRNA-specific primers, or with primers to detect the control transcript *U6* snRNA, along with a universal primer. *U6* snRNA was used for normalization of exosomal miRNA (Xu et al., 2017; Zhang et al., 2018).

### Human RNA-binding domain and protein microarray analysis

This assay was performed according to modifications of a previously described method (Chen et al., 2020). GST recombinant proteins were designed to include reported and suspected RNA-binding domains and synthesized by Biomatik Corporation in the pGEX-4T1 vector (Amersham). Recombinant proteins were expressed and purified using the GST-fusion system. GST proteins were then arrayed, in duplicate, using an Aushon 2470 Microarrayer (Quanterix) onto nitrocellulose-coated glass slides (Grace Bio-Labs). The RNA-binding domains array contains approximately 354 GST recombinant proteins, which include SAP, RRM, KH, DSRM and YTH domains. Full-length GST- and His-fusion proteins were printed, in duplicate, onto nitrocellulose-coated glass slides.

To screen for potential RNA–protein interactions, the RNA-binding domains array was probed with biotinylated-miRNA oligonucleotides. miRNA oligonucleotides were designed with 5′ biotinylation and a 3′ Cy5 fluorophore and synthesized by Integrated DNA Technologies. Microarrays were blocked for 1 h with RNA-binding buffer (20 mM Tris-HCl at pH 7.4, 200 mM KCl, 0.2 mM EDTA, 0.05% NP40, 2 µg/µl heparin). miRNA oligonucleotides were prepared for microarray probing by incubating miRNA at 1 µM with streptavidin–Cy3 in RNA-binding buffer in presence of 0.4 U RNase inhibitor. Primed miRNA oligonucleotides were then incubated with microarrays overnight at 4°C in RNA-binding buffer with RNase inhibitor. Microarrays were dried and scanned using the InnoScan 1100 AL Fluorescence Scanner (Innopsys) and intensities were quantified using Mapix software (Innopsys).

The Invitrogen Ultimate 16,813 ORF collection, subcloned into a yeast expression vector that allows for galactose-dependent overexpression of N-terminal GST- and 6× His-tagged recombinant proteins, was spotted onto Fullmoon slides (Fullmoon Biosystem) using the NanoPrint LM210 system (ArrayIT) as previously described (Hu et al., 2009). The quality of these protein microarray chips, along with that of control proteins (such as IgG, GST and histones), was assayed using a randomly selected slide from the same batch, and at least 89% of the proteins were expressed correctly.

To identify miRBPs, the protein microarray chips were equilibrated and washed three times for 15 min with RNase-free PBS at pH 7.0 with 2 U/μl RNase inhibitor SUPERase-In (Thermo Fisher Scientific). The proteins on the chips were blocked with filtered 3% high quality IgG-/protease-free BSA (Jackson ImmunoResearch Laboratory) for 1 h at room temperature, then washed three times with RNase-free PBS buffer (filtered RNase-free PBS pH 7.0 with 1 U/μl RNase inhibitor). The protein microarray chips were incubated with internally Cy5- or DY647-labeled let-7b, miR-21, and miR-130b (100 ng) for 1 h, wrapped in aluminum foil on a shaking platform at 50 rpm. The chips were washed five more times with RNase-free PBS buffer for 10 min.

For detection of all possible proteins, the chips were then incubated with rabbit anti-GST antibody (71-7500, Invitrogen) at 1:5000 for 1 h at room temperature, washed three times for 15 min in RNase-free PBS buffer, and incubated with 1:1000 Cy3 goat anti-rabbit IgG antibody (A10520, Invitrogen) in RNase-free PBS buffer for 20 min at room temperature. The final washing was performed three times for 15 min in RNase-free PBS buffer and rinsed once in double-distilled H_2_O. Protein microarray chips were dried via centrifugation at 200 ***g*** for 2 min using a 50 ml conical tube.

The chips were scanned with GenePix 4000B (Axon Instruments) for quantification and statistical analysis. Fluorescence from bound Cy5-/DY647-labeled let-7b, miR-21, and miR-130b was measured at 635 nm, and from Cy3-labeled anti-GST at 532 nm. Signal intensity values for each spot were obtained by scanning to find the ratio of foreground to background signals, and normalized with GST signal intensity. The mean and standard deviation of signal intensity of all proteins on the chip were calculated using R package, and *P*≤0.05 was considered statistically significant.

### Gene Ontology analysis

The list of 60 identified let-7b-binding proteins was submitted to the online DAVID functional annotation resource (v6.8) (Huang et al., 2009a,b), where the overrepresented proteins were identified and compared with the human proteome database.

### EV preparation

EV isolation was performed as previously described (Kang et al., 2024; Kwon et al., 2016). Briefly, the cells were cultured with a freshly replaced serum-free medium. After 30 h, the conditioned medium containing EVs was collected and centrifuged at 500 ***g*** for 20 min. The supernatant was then incubated with 1 U/ml of benzonase (Millipore Sigma) for 15 min at room temperature and subsequently centrifuged at 2000 ***g*** for 20 min at 25°C. The obtained supernatant was then subjected to centrifugation at 10,000 ***g*** for 1 h at 25°C and subsequently filtered through a 0.2-μm asymmetric polyethersulfone membrane (Millipore Sigma). The filtered supernatant underwent tangential flow filtration using a 500-kDa cutoff Biomax cassette (Millipore Sigma). The feed rate was set to 4–5 ml/min with a poly pump (Buchler), ensuring that inlet and operating pressures remained below 68,947.6 Pa and 24,131.66 Pa, respectively. The retained particles were then washed with Dulbecco's phosphate-buffered saline (DPBS, Thermo Fisher Scientific). The volume of the final processed sample was typically concentrated 10- to 15-fold. The EV-containing retentate was subsequently centrifuged at 200,000 ***g*** using a SW60 Ti rotor (Beckman Coulter) for 1 h at 4°C. After resuspending the pellet in DPBS, the isolated EVs were stored at −80°C for future use. The size and number of EVs were measured using nanoparticle tracking analysis, and samples were stored at −20°C.

### Structure modeling

The crystal structure of QKI (PDB code 4JVH; Teplova et al., 2013) was used to calculate suitable binding conformations and associated binding affinities for all possible six-nucleotide subsequences of let-7b, miR-21 and miR-130b. Molecular dockings were carried out by the program PyRx Virtual Screening Tool Autodock Vina v0.9.7 (https://vina.scripps.edu/) for every RNA fragment, five times per model, with the determination of minimum docking energy among 15 calculated values. To run the software, the PDB file was converted to PDBQT format via OpenBabelGUI program (https://openbabel.org/docs/GUI/GUI.html). The docking models were presented by PyMOL software (The PyMOL Molecular Graphics System, v2.0 Schrödinger, LLC.).

### Fluorescence polarization assay

To verify the RNA-binding affinity of QKI with let-7b, miR-21, miR-130b, let-7b mutant and N20 random RNA, recombinant QKI was incubated with 3 nM Cy3-labeled RNAs in assay buffer (20 mM HEPES pH 7.4, 150 mM NaCl, 2 mM MgCl_2_, 1 U/µl RiboLock RNase inhibitor and 10 mM dithiothreitol) at room temperature. The assay was performed in 96-well non-binding black plate (Corning), with fluorescence polarization measured using a Spectramax iD5 microplate reader (Molecular Devices), with excitation and emission wavelengths at 530 nm and 570 nm, respectively. Millipolarization units (mP) were used to express fluorescence polarization values defined by the equation mP=1000×[(I_II_−I_⊥_)/(I_II_+I_⊥_)], where I_II_ and I_⊥_ are parallel and perpendicular emission intensity measurements, respectively. The data were corrected for background (RNA sample alone) and G-factor, then fitted by a one-site binding model using the equation y=y_0_+(y_max_x/K_d_+x), where y is the corrected fluorescence polarization, y_0_ is the polarization value without protein, y_max_ is the maximum binding fluorescence polarization signal, x is the protein concentration, and K_d_ is the dissociation constant. x and y_max_ were used as fitting parameters and nonlinear regression was performed using GraphPad Prism. Measurements were taken after 5 min incubation between the protein and RNA at room temperature.

### Immunofluorescence

All manipulations were performed with Institutional Animal Care and Use Committee (IACUC) approval from MD Anderson Cancer Center. Whole brains were collected from *Qki^+/+^* and *Qki^−/−^* using *Nestin-* or *Plp-Cre* driver mice (Shingu et al., 2017). The frozen brain tissues were sliced and the resulting slides were post-fixed in 1× PBS containing 4% paraformaldehyde for 10 min, permeabilized with PBS containing 0.3% Triton X-100 (0.3% PBST) for 10 min, and then blocked with 0.3% PBST containing 10% bovine serum albumin (BSA) for 1 h at room temperature. The slides were then incubated with the corresponding primary antibodies overnight at 4°C. All antibodies were diluted 1:1000 in 0.3% PBST. The following primary antibodies were used: mouse anti-NeuN (Millipore, MAB377), rabbit anti-QKI5 (Bethyl Laboratories, IHC-00574), mouse anti-CNPase (Millipore, MAB326), rabbit anti-CNPase (Santa Cruz Biotechnology, sc-30158), mouse anti-GFAP (Millipore, MAB360), goat anti-GFAP (Santa Cruz Biotechnology, sc-6170), mouse anti-Iba1 (Millipore, MABN92), rabbit anti-Iba1 (Wako, 019-19741), rabbit anti-TLR7 (Novus Biologicals, NBP2-24906), mouse anti-J2 (SCICONS, 10010500, Hungary), rabbit anti-NF (BioLegend, 841001), rabbit anti-TLR3 (NB100-56571, Novus Biologicals) and mouse anti-pan-QKI (NeuroMab, 75-168, Davis, CA, USA). After primary antibody incubation, the slides were washed with PBS and incubated with Alexa Fluor 488- or 594-conjugated secondary antibodies (a21206 or a21203, Life Technologies) diluted in 0.3% PBST (1:1000) for 2 h at room temperature. After washing with PBS, to observe the nucleus, the slides were counterstained with 4′,6-diamidine-2′-phenylindole dihydrochloride (DAPI, Roche, 10236276001, Germany). The slides were then washed again with PBS, mounted and analyzed by confocal microscopy (LSM700; Carl Zeiss, Germany). Anti-pan-QKI antibody (NeuroMab, 75-168) was generated by using amino acids 1–341 (full-length) of human QKI5, which shares identity with mouse Qki (100%, 341/341 amino acids identical) and rat Qki (99%, 339/341 amino acids identical). The anti-Qki5 antibody was generated by using Qki5 C-terminal exon 8 (identical in human and mouse), which does not exist in Qki6, Qki7 and Qki7b) (Thermo Fisher Scientific, IHC-00574, previously from Bethyl Laboratories).

### Chromatin immunoprecipitation

Chromatin immunoprecipitation assay was performed using SH-SY5Y cells treated with EVs isolated from conditioned media of either control or QKI shRNA-treated HeLa cells. For cross-linking, the cells exposed to EVs were treated with 1% formaldehyde for 10 min at room temperature. Crosslinking reactions were quenched with 125 mM glycine for 5 min at room temperature. The cells were washed with PBS twice and centrifuged at 1000 ***g*** for 5 min. After centrifugation, cell pellets were lysed in IP buffer composed of 0.1% SDS, 1% Triton X-100, 0.1% sodium deoxycholate and 140 mM NaCl containing protease inhibitors. The cell lysates were sonicated for 10 s with 10 s interval four times. Sheared chromatin was incubated with either 3 µg anti-IRF7 antibody (Santa Cruz Biotechnology, sc-74472) or normal IgG antibody (Santa Cruz Biotechnology, sc-2025) conjugated with protein A/G-Sepharose beads (#20349, Thermo Fisher Scientific) overnight at 4°C. After the beads were washed five times with wash buffer composed of 0.1% Triton-X, 20 mM Tris (pH 8.0), 150 mM NaCl and 1 mM EDTA, the beads were incubated with 10 unit/µl RNase A and 300 mM NaCl for 4 h at 65°C. Then, the beads were further incubated with proteinase K digestion buffer composed of 50 mM Tris-HCl (pH 8.0), 10 mM EDTA and 0.4 mg/ml Proteinase K for 2 h at 65°C. The immunopellets were subjected to phenol/chloroform/isoamyl alcohol (25:24:1) and the purified DNA was assayed with qPCR using the primers amplifying *IFNA1* gene promoter sequences. Data were normalized to input and analyzed relative to the nonspecific IgG control.

### Affinity capture and detection of 5-EU-labeled RNA

*De novo*-synthesized let-7b miRNA via EVs was tracked using the Click-iT Nascent RNA Capture Kit (Invitrogen, C10365). Briefly, to label let-7b miRNA, QKI-depleted or control donor cells (HeLa) were treated with 0.28 mM 5-EU (E10345, Invitrogen) for 24 h and EVs containing 5-EU-labeled RNA were isolated from the conditioned media. The EVs were added to culture medium of SH-SY5Y cells and the cells were grown for 6 h; then, the total RNA of recipient cells was isolated by TRIzol (Invitrogen, 15596026) according to the manufacturer's protocol. For purification of 5-EU-labeled RNA, 5 µg of total RNA and 0.5 mM biotin-azide (B10184, Invitrogen) was used for RNA biotinylation, and precipitated with 7.5 M ammonium acetate in combination with ice-cold 100% ethanol. The biotinylated RNAs were subsequently incubated with streptavidin-conjugated beads for 1 h at room temperature with gentle agitation. Then, the beads were washed eight times with supplied wash buffer according to the manufacturer's instructions. The 5-EU-labeled RNAs on beads were extracted with elution buffer composed of 20 mM Tris (pH 7.4) and 1 mM EDTA for 4 min at 92°C, and the labeled let-7b was determined by qPCR in comparison with the standard condition from which EVs were isolated without 5-EU labeling and applied to the recipient cells.

### Protein interactome analysis

Protein–protein interactions were sourced from BioGRID, build 4.3.194 (Stark et al., 2006). Networks were initially created using R software to select the appropriate protein–protein interactions from hit proteins and BioGRID data. Output tables were uploaded into Cytoscape 3.8.0 to create visual figures. Previous ‘known’ hits are from a previous publication (Treiber et al., 2017).

## Supplementary Material



10.1242/joces.261575_sup1 Supplementary information

Table S1. List of RNA-binding proteins and intensities of the bound/labeled miRNA

Table S2. List of proteins and intensities of the bound/labeled miRNA

Table S3. Gene ontology analysis proteins identifed from human protein arrays

Table S4. DNA and RNA sequences used in this study
