# Supplementary material for: Mature microRNA-binding protein QKI suppresses extracellular microRNA let-7b release
Source: J Cell Sci. 2024 Nov 6;137(21):jcs261575. doi: 10.1242/jcs.261575 (PMC11574364; doi:10.1242/jcs.261575)
Supplement: Supplementary information [file joces-137-261575-s1.pdf]

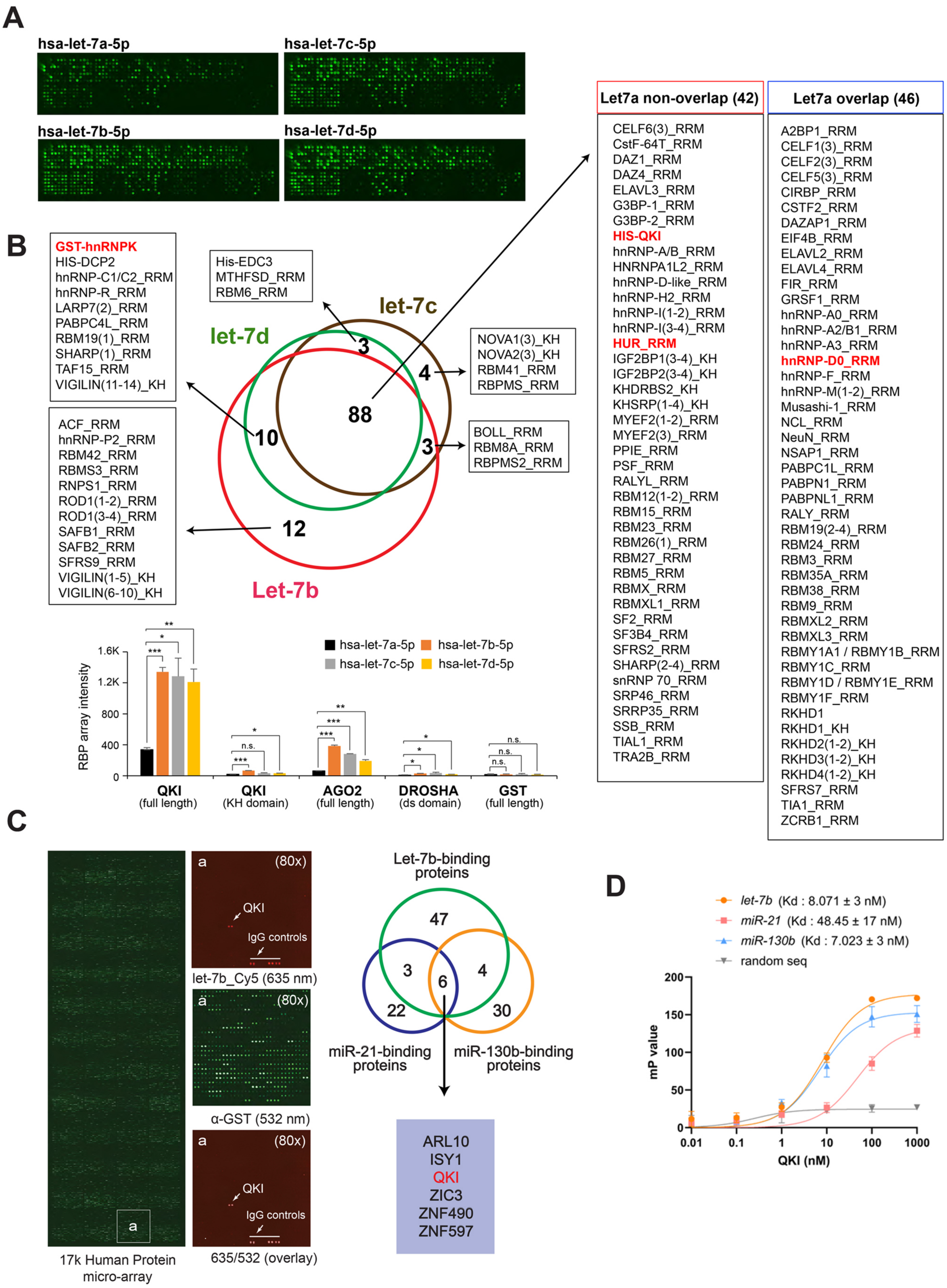

Journal of Cell Science • Supplementary information

Fig. S1. High throughput protein array profiled RBPs directly binding with mature miRNA let-7.

(A) Fluorescence images of biotinylated let-7a, 7b, 7c and 7d bound to proteins spotted on the microarray slides.

(B) Venn diagram of RBPs commonly identified from protein array (>500 intensity) followed by list of RBPs in each overlaps. Intensity of let-7a, 7b, 7c, and 7d were plotted using values from full length QKI, its KH domain, AGO2, DROSHA and GST as a negative control. N = 2, ***p < 0.001, **p < 0.01, *p < 0.05, n.s., not significant from Student's t-test.

(C) Protein array was performed with human let-7b containing internal Cy5 labeling on the 18th uridine residue, miR-21 containing DY647 on the 18th guanosine, or miR130b. The resulting fluorescence was detected from Cy5 (or DY647) at 635nm, and Cy3-labeld anti-mouse IgG antibody (532 nm) against GST. The six proteins identified from all three protein arrays are listed. The results are an average of two independent experiments.

(D) Anisotropy analysis of recombinant QKI protein with let-7b, miR-21, miR-130b and N20 random sequence (random seq). The results are average of three independent experiments with standard deviation.

Journal of Cell Science • Supplementary information

A let-7b pull down

Gene symbol	Score
HNRNPA2B1	41.06
HNRNPH1	39.36
GRSF1	26.94
HNRNPM	26.15
HNRNPU	25.84
HNRNPH2	25.42
CNBP	24.39
PC	23.2
ACACA	21.08
PLEC	20.65
KRT1	20.39
HNRNPA1	18.54
NCL	15.79
YBX1	14.98
KRT9	14.34
KHSRP	13.27
TARDBP	11.9
HNRNPF	11.22
HNRNPR	10.04
YBX3	10.02
CELF1	9.11
TUBB	8.39
FUS	8.36
DAZAP1	8.2
HNRNPA0	8.08
COLGALT1	7.98
TUBB4A	7.97
HNRNPK	7.95
HSPD1	7.9
PABPC1	7.77
HNRNPUL1	7.76
ALYREF	6.92
TUBA1C	6.52
HSPA8	6.31
ALB	6.31
PCCA	5.77
RPL12	5.33
DDX3Y	5.23
HNRNPH3	5.12
PCCB	4.98
HIST1H1C	4.73
MRPS28	4.59

B let-7i pull down

Gene symbol	Score
HNRNPA2B1	43.87
PC	29.26
HNRNPM	27.13
KRT1	21.12
KRT10	18.01
KHSRP	17.81
HNRNPU	17.29
KRT9	12.13
ALYREF	10.85
YBX1	9.66
ACACA	9.57
HNRNPA0	9.11
FUBP1	8.02
HNRNPR	7.19
HSPA9	7.07
MRPL15	6.85
KRT2	6.61
HNRNPA1	6.42
HNRNPUL1	6.23
HNRNPC	6.12
PUF60	5.16
PCCA	5.07
MCCC1	4.84
NCL	4.76
ZRANB2	4.73
PABPC1	4.69
DHX36	4.29
HNRNPK	4.16

C miR-141c pull down

Gene symbol	Score
NCL	168.2
GRSF1	120.2
MCCC1	90.3
MAPK8IP3	30.1
FBN2	20.1
NOL7	20.1
PARVA	20.1
SPTBN1	20.1
TNF	18.1
OTOF	18.1
DEF	16.7
USP32	16.4
FGFBP3	16.2
SMURF1	14.2
KIAA0564	14.1
NUMA1	12.7
CCDC123	12.6
SRRT	12.2
THEMIS	10.2
HNRPAB	10.2
FOXC1	10.1
PHF11	10.1
C3	10.1
FLNC	10.1
EIF2B1	10.1
TIMM50	10.1
FBN2	10.1
MACF1	10.1
KDM5B	10.1
CTAG1A	10.1
PRPF39	10.1

D miR-200 pull down

Gene symbol	Score
NCL	416.3
GRSF1	290.5
NCL	258.2
SRSF1	90.2
MCCC1	40.3
SSB	40.2
TSNAX	30.2
GRSF1	30.2
HNRNPF	30.2
TIMM50	26.2
SBNO2	20.3
TUBB3	20.2
THEMIS	20.2
HNRPH1	20.2
CTAG1A	20.1
HSP90B1	20.1
MAGI3	20.1
CALML3	18.1
SENP6	18.1
CENPI	16.6
CCDC29	16.5
CCDC154	16.4
UTP20	16.2
STXBP4	16.2
TRIM8	16.2
AHCTF1	16.1
TULP1	16.1
ABCC8	16.1
DEF	14.7
ISY1	14.3
GGT5	14.2
HECTD1	12.2
PARP1	10.6
DNAH2	10.3
SFRS6	10.3
ARHGAP12	10.3
ABR	10.3
MYH2	10.2
TIMM50	10.2
CCDC88A	10.2
ITGAL	10.2
CIZ1	10.2
POSTN	10.2
ZNF540	10.2
GRSF1	10.2
C14orf73	10.2
CTNNA1	10.2
CDK11A	10.1
FOXC1	10.1
FUT2	10.1
ZNF574	10.1
MAPK8IP3	10.1
SLFN5	10.1
C4B	10.1
EIF2B1	10.1
LCT	10.1
CAND2	10.1
CHD1	10.1
TMEM8C	10.1
FOXC1	10.1
TNF	10.1
HAUS6	10.1
TARBP1	10.1
RBM41	10.1
MDN1	10.1
PDE12	10.1
PTPRC	10.1
DNAH2	10.1
LGALS12	10.1
LMNB1	10.1
FSIP2	10.1
FAM43A	10.1
PKDREJ	10.1
FBN2	10.1
BSG	10.1
RASSF9	10.1
1-Mar	10.1
TNFAIP2	10.1
AGFG2	10.1
FBN2	10.1
AMY1A	10.1
ATAD3B	10.1
CRTC3	10.1
CCR10	10.1
XRCC4	10.1
LSM4	10.1
CSHY	10.1
CDADC1	10.1

E

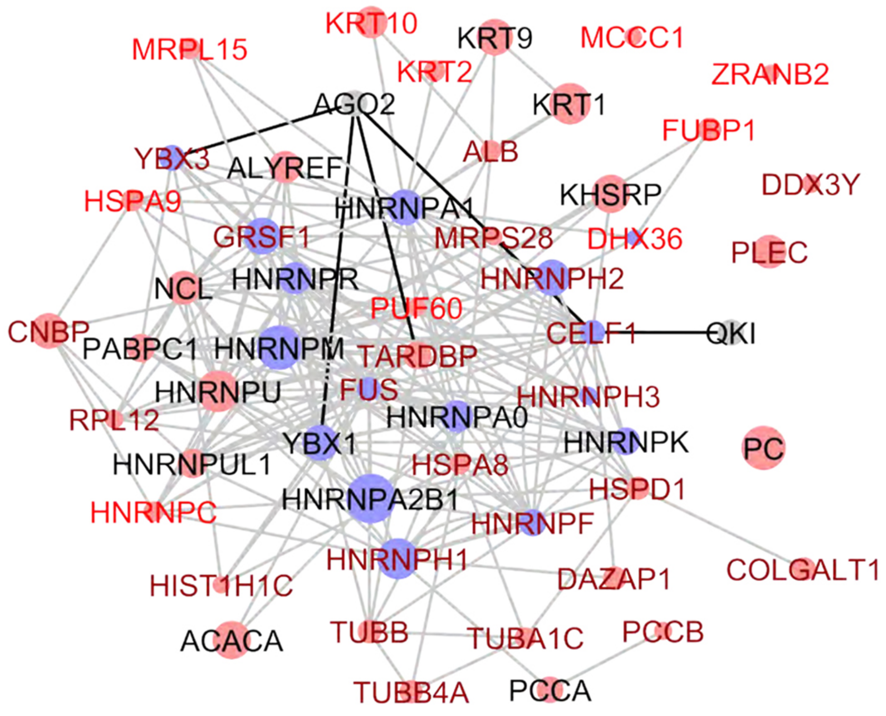

Fig. S2. Mass Spectrometry of proteins identified from mature miRNA pull down.

(A-D) List of proteins profiled from proteins after affinity pull down of let-7b, let-7i, miR-141c, or miR-200.

(E) Protein-protein interactions of hits identified in proteomic analysis. Edges are derived from BioGRID. Blue fill indicates previous identification of miRNA-binding proteins specific to either miRNA. Red fill indicates novel identified miRNA-binding proteins. Gene text color indicates whether the gene protein product was found bound to let-7i only (bright red), let-7b only (dark red), or both (black). Node size is proportional to signal (let-7b and 7i) in Mass Spectrometry.

Journal of Cell Science • Supplementary information

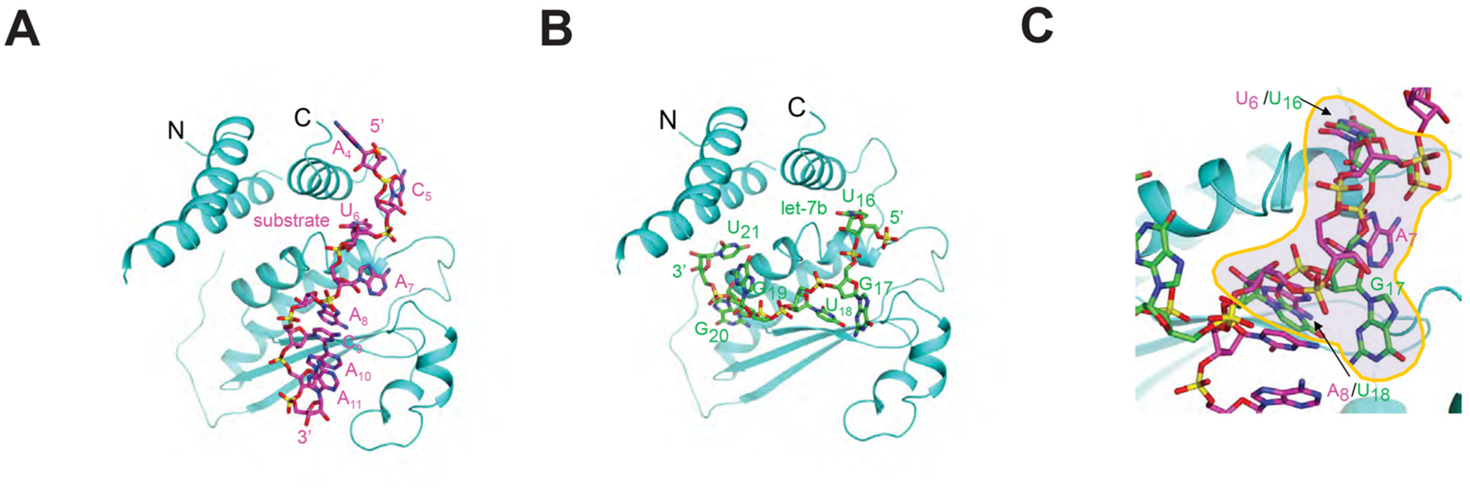

D

RNA sequence (5'→3')	Affinity (kcal/mol)
UGUGGU	-9.8
UAGUAG	-9.6
UAGGUU	-9.3
GUAGGU	-9.1
UUGUAU	-8.7
GAGGUA	-8.5
GUAGUA	-8.2
GUGGUU	-8.0
UGUAUA	-8.0
UGAGGU	-7.6
UAUAGU	-7.6
AUAGUU	-7.4
GUAUAG	-7.4
AGUAGG	-7.1

*Substrate ACUAACAA
**let-7b UGAGGUAGUAGGUUGUGUGGUU

E

RNA sequence (5'→3')	Affinity (kcal/mol)
CAGACU	-9.4
CUUAUC	-8.8
UCAGAC	-8.3
ACUGAU	-8.2
UGAUGU	-8.1
UUAUCA	-7.5
UAGCUU	-7.4
UGUUGA	-7.0

*Substrate ACUAACAA
**miR-21 UAGCUUAUCAGACUGAUGUUGA

F

RNA sequence (5'→3')	Affinity (kcal/mol)
ACAUUG	-8.9
UGUCUG	-8.7
GUCUGC	-8.6
ACUGUC	-8.3
UUCACA	-8.1
UGCUAC	-7.9
CUACUG	-7.4
UGUGCU	-6.9

*Substrate ACUAACAA
**miR-130b UUCACAUUGUGCUACUGUCUGC

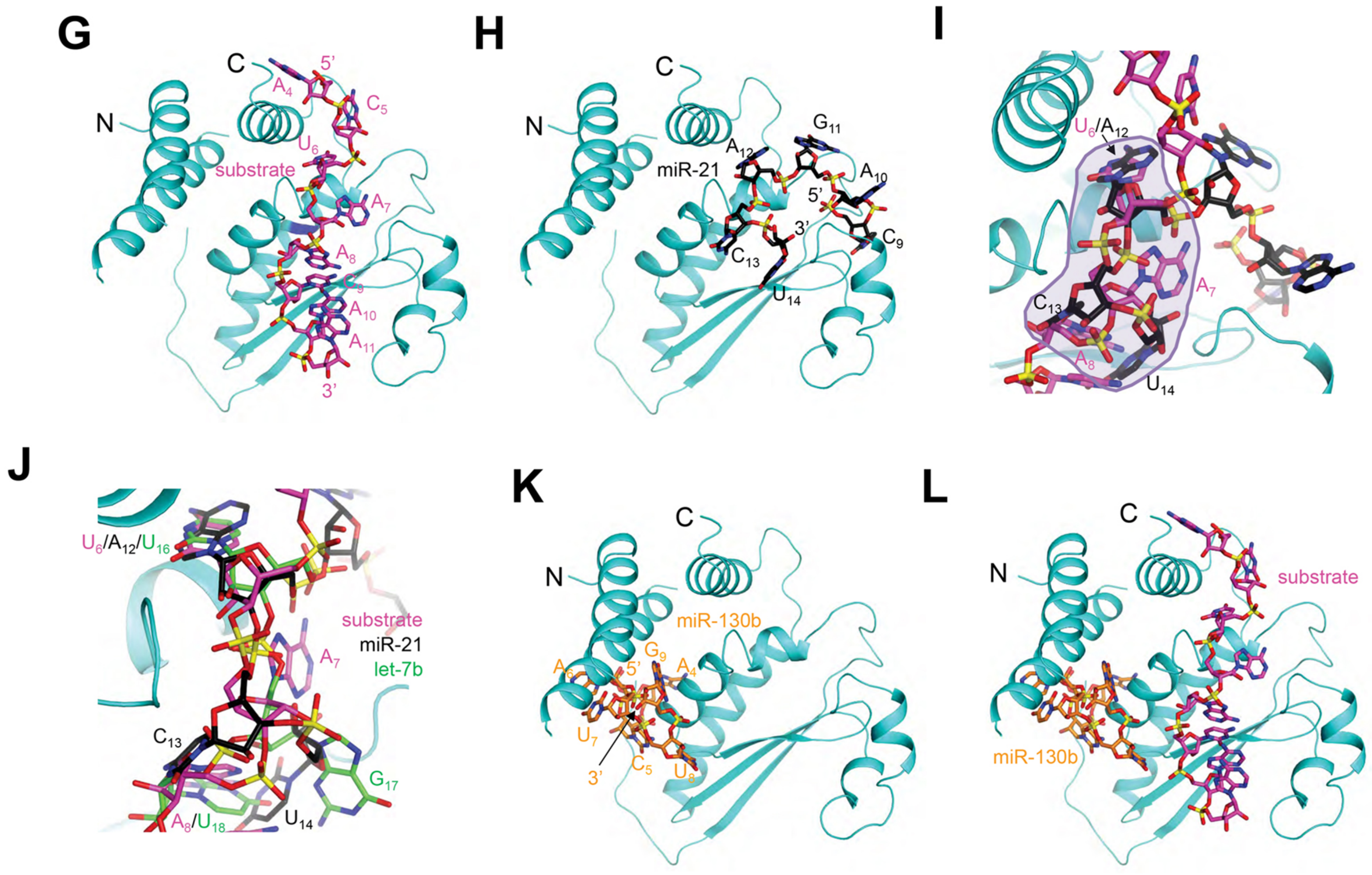

Fig. S3. Structural modeling of miRNA fitting into QKI.

(A) Crystal structure of QKI-substrate complex (pdb code: 4JVH); substrate RNA shown in magenta.

(B) Simulated model of QKI in complex with a partial sequence of let-7b (UGUGGU); let-7b RNA shown in green.

(C) Overlaid structure of the substrate and let-7b; signature motif UAA of the substrate shown in orange.

(D-F) Calculated binding affinity values of varied partial sequences of miRNAs. let-7b, miR-21 and miR-130b were simulated for modeling using PyRx Virtual Screening Tool Autodock Vina Version 0.9.7.

(G-L) Simulated models of QKI in complex with a partial sequence of let-7b (UGUGGU), miR- 21 and miR-130b. The let-7b RNA shown in green. Overlaid structure of the substrate and let-7b. The signature motif UAA of the substrate shown in orange.

Journal of Cell Science • Supplementary information

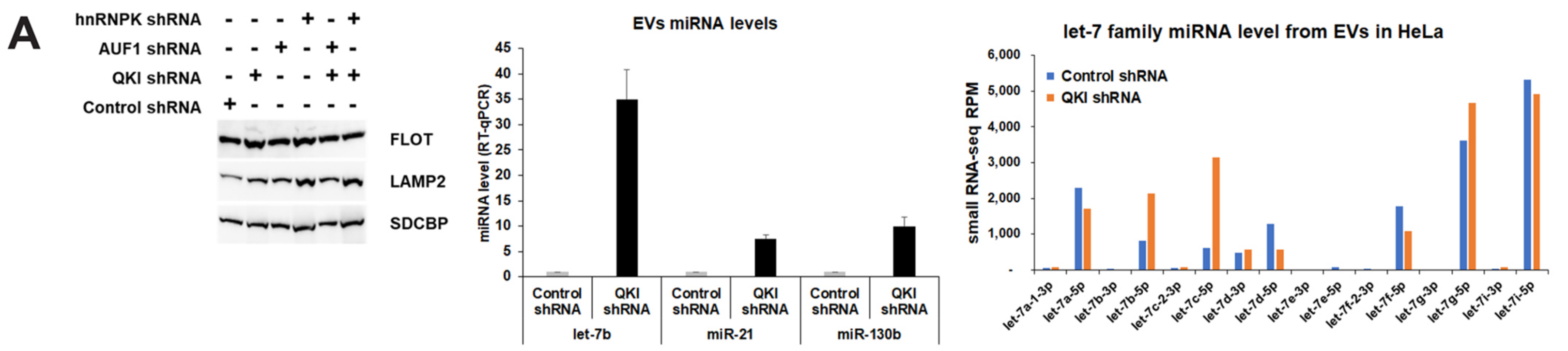

B

Samples	Total number (ea/mL)	Size mean (nm)	Standard Deviation	D10 (nm)	D50 (nm)	D90 (nm)
Control shRNA	5.05 e+11	187.9	69.3	111.6	183.9	293.6
QKI shRNA	2.97 e+11	183.7	83.1	90.1	168.8	266.1
AUF1 shRNA	6.32 e+11	176.1	65.5	110.2	167.4	241.9
hnRNPK shRNA	4.10 e+11	183.7	71	98.2	178.6	264.2
QKI and AUF1 shRNA	4.31 e+11	197.4	84.2	107.3	183.4	315.3
QKI and hnRNPK shRNA	2.31 e+12	178.4	65.8	98.3	174.1	262.5

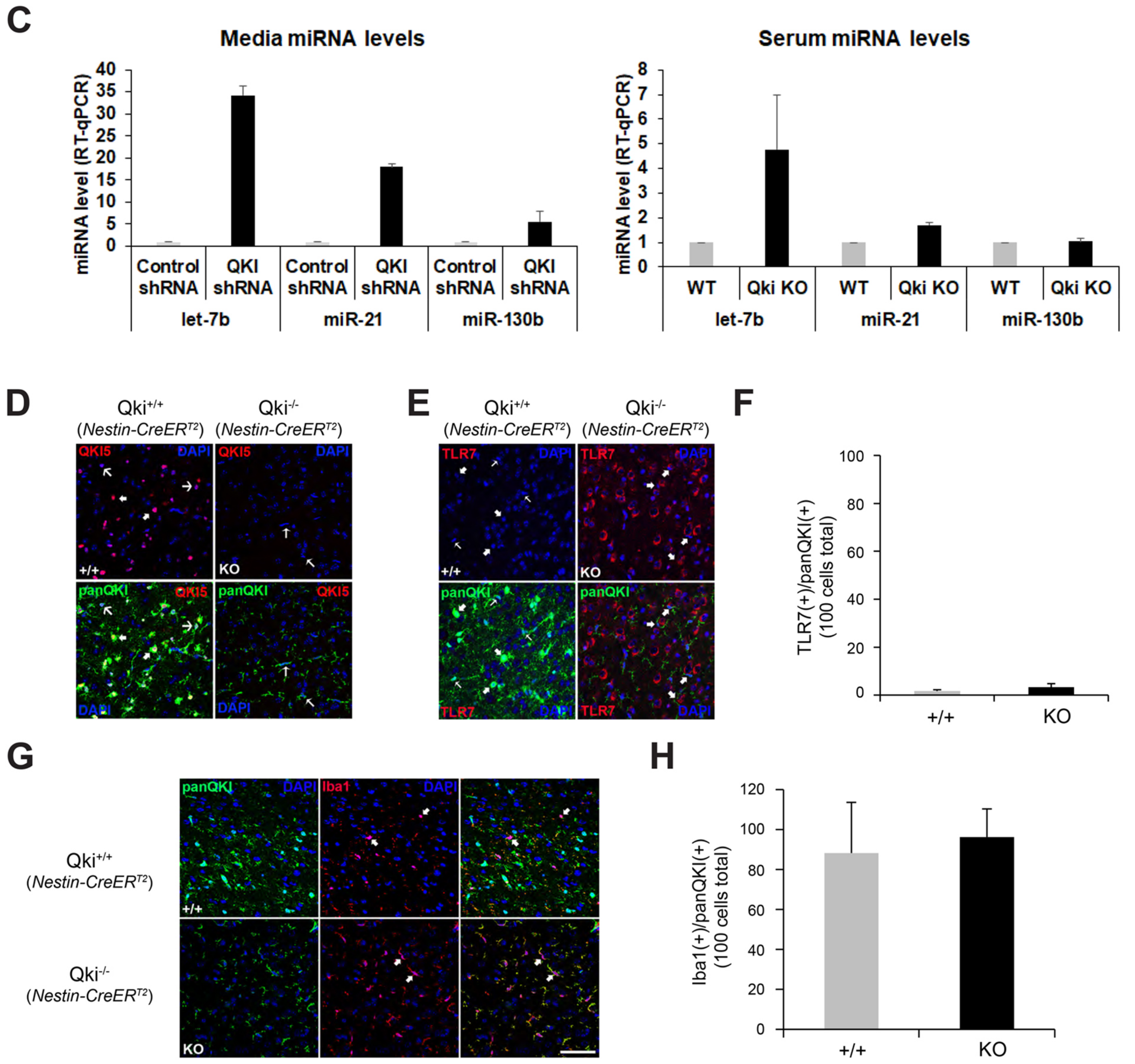

Fig. S4. Extracellular Vesicle purification and expression of pan-Qki in neurons and microglia.

(A) Immunoblots of extracellular vesicles marker proteins after transfection of plasmids expressing shRNAs targeting QKI, AUF1, hnRNPK or GFP as a control. The immunoblot images are representative of three independent experiments. RT-qPCR levels of miRNAs purified from the corresponding extracellular vesicles. The graphs are averages and standard deviation of three independent experiments. RNA-seq level (reads per million, RPM) of let-7 family miRNAs purified from extracellular vesicles of QKI or Control shRNA.

(B) Size quantification of extracellular vesicles used in (A).

Journal of Cell Science • Supplementary information

(C) RT-qPCR levels of miRNAs purified from HeLa media (left) and mouse serum (right). The graphs are averages and standard deviation of three independent experiments.

(D, E, G) Confocal images show immunolabeling for QKI5, TLR7 or Iba1 (red) with panQKI (green), using sect ions of Nestin-CreERT2 Qki$_{+/+}$ and Qki KO mice. Scale bar = 50 µm. In Qki$_{+/+}$, thick and thin arrows of indicate oligodendrocytes and astrocytes. In Qki$_{-/-}$, arrows indicate microglia. N = 3.

(F, H) Quantitative data indicates counts of TRL7 or Iba1/panQKI-double positive cells among 100 DAPI-positive profiles in Nestin-CreERT2 Qki mice. N = 3.

DAPI-positive profiles in Nestin-CreER^{T2} Qki mice. N = 3.

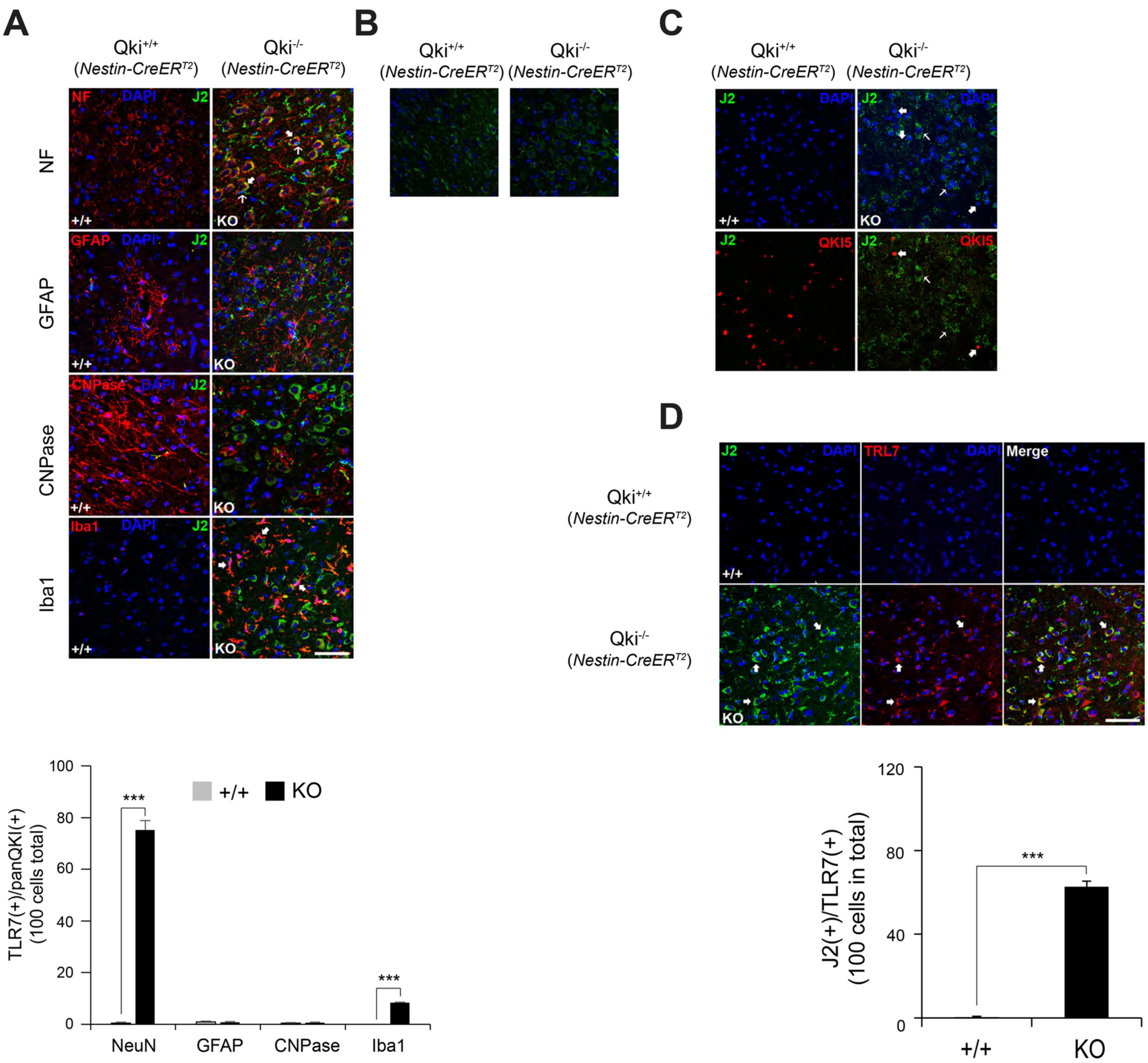

Fig. S5. Expression of dsRNA and Qki5 in neurons, astrocytes, oligodendrocytes and microglia in mouse cerebral cortex.

(A) Confocal images show immunolabeling for dsRNA using J2 antibody (green) with NF (red), GFAP, CNPase, and Iba1 from sections of Nestin-CreER^{T2} Qki$^{+/+}$ and Qki KO mice. In NF staining, thick and thin arrows indicate J2-positive cells and microglia surrounding J2-positive cells. In Iba1 staining, thick arrows indicate microglia surrounding J2-positive cells. Scale bar = 50 µm. J2-positive cells were counted with J2 immunolabeling out of 100 DAPI-positive cells in Nestin-CreER^{T2} Qki mice. N = 3, ***p < 0.001, from Student's *t*-test.

(B) Confocal images show immunolabeling for TLR3 (green) from sections of Nestin-CreER^{T2} Qki$^{+/+}$ and Qki KO mice.

(C, D) Confocal images show immunolabeling for J2 (green) with QKI5 (red) or TLR7, using sections of Nestin-CreER^{T2} Qki$^{+/+}$ and Qki KO mice. Scale bar = 50 µm. In C, thick and thin arrows indicate J2-positive and -negative cells. In D, thick arrows indicate J2/TLR7-double positive cells. Quantitative data indicates counts of J2/TRL7-double positive cells among 100 DAPI-positive profiles in Nestin-CreER^{T2} Qki mice. N = 3, ***p < 0.001, from Student's *t*-test.

Journal of Cell Science • Supplementary information

Table S1. List of RNA-binding proteins and intensities of the bound/labeled miRNA

Available for download at
https://journals.biologists.com/jcs/article-lookup/doi/10.1242/jcs.261575#supplementary-data

Table S2. List of proteins and intensities of the bound/labeled miRNA

Available for download at
https://journals.biologists.com/jcs/article-lookup/doi/10.1242/jcs.261575#supplementary-data

Table S3. Gene ontology analysis proteins identifed from human protein arrays

Available for download at
https://journals.biologists.com/jcs/article-lookup/doi/10.1242/jcs.261575#supplementary-data

Table S4. DNA and RNA sequences used in this study

Available for download at
https://journals.biologists.com/jcs/article-lookup/doi/10.1242/jcs.261575#supplementary-data

Journal of Cell Science • Supplementary information
