# Supplementary material for: Mature microRNA-binding protein QKI suppresses extracellular microRNA let-7b release
Source: J Cell Sci. 2024 Nov 6;137(21):jcs261575. doi: 10.1242/jcs.261575 (PMC11574364; doi:10.1242/jcs.261575)
Supplement: Supplementary file 1 [file joces-137-261575_review_history.pdf]

Mature microRNA-binding protein QKI suppresses extracellular microRNA let-7b release

Kyung-Won Min, Kyoung-Min Choi, Hyejin Mun, Seungbeom Ko, Ji Won Lee, Cari A Sagum, Mark T Bedford, Young-Kook Kim, Joe R Delaney, Jung-Hyun Cho, Ted M Dawson, Valina L Dawson, Waleed Twal, Dong-Chan Kim, Clarisse H Panganiban, Hainan Lang, Xin Zhou, Seula Shin, Jian Hu, Tilman Heise, Sang-Ho Kwon, Dongsan Kim, Young Hwa Kim, Sung-Ung Kang, Kyungmin Kim, Sydney Lewis, Ahmet Eroglu, Seonghyun Ryu, Dongin Kim, Jeong Ho Chang, Junyang Jung and Je-Hyun Yoon
DOI: 10.1242/jcs.261575

Editor: Giampietro Schiavo

Review timeline
Original submission: 31 August 2023
Editorial decision: 31 October 2023
First revision received: 4 July 2024
Editorial decision: 1 August 2024
Second revision received: 23 August 2024
Accepted: 3 September 2024

Original submission

First decision letter

MS ID#: JOCES/2023/261575

MS TITLE: Mature microRNA-binding protein QKI suppresses extracellular microRNA release

AUTHORS: Kyung-Won Min, Seungbeom Ko, Ji Won Lee, Hyejin Mun, Cari A Sagum, Mark T Bedford, Young-Kook Kim, Joe R Delaney, Jung-Hyun Cho, Ted M Dawson, Valina L Dawson, Waleed Twal, Dong-Chan Kim, Clarisse H Panganiban, Hainan Lang, Xin Zhou, Seul A Shin, Jian Hu, Tilman Heise, Sang-Ho Kwon, Dongsan Kim, Young Hwa Kim, Sung-Ung Kang, Jeong Ho Chang, Junyang Jung, and Je-Hyun Yoon

We have now reached a decision on the above manuscript.

To see the reviewers' reports and a copy of this decision letter, please go to: https://submit-jcs.biologists.org and click on the 'Manuscripts with Decisions' queue in the Author Area. (Corresponding author only has access to reviews.)

As you will see, the reviewers raise several substantial criticisms that prevent me from accepting your manuscript. The type and number of additional information and requested changes go well beyond those traditionally associated with a revision. However, the reviewers suggest that a revised version might prove acceptable, if you can address their many concerns. Whilst I would fully understand if you opt to resubmit your manuscript elsewhere, I would in principle be happy to see a revised manuscript if you can deal satisfactorily with the criticisms on revision, We would then return it to the reviewers.

Please ensure that you clearly highlight all changes made in the revised manuscript. Please avoid using 'Tracked changes' in Word files as these are lost in PDF conversion.

© 2024. Published by The Company of Biologists under the terms of the Creative Commons Attribution License (https://creativecommons.org/licenses/by/4.0/).

I should be grateful if you would also provide a point-by-point response detailing how you have dealt with the points raised by the reviewers in the 'Response to Reviewers' box. Please attend to all of the reviewers' comments. If you do not agree with any of their criticisms or suggestions please explain clearly why this is so.

Reviewer 1

Advance summary and potential significance to field

This is an interesting, but a descriptive study. The abstract could be rephrased to make it clearer. Discussion must be revised.

Comments for the author

Major Points:
1. Abstract is unclear. State what is the goal of this research and what is new here.
2. This is a decsriptive and preliminary study, not mechanistic.
3. Discussion is too short. In the discussion, discuss your results focusing on what is new here, in other words, synthesize new knowledge through discussion.

Reviewer 2

Advance summary and potential significance to field

In this manuscript, the authors reported a novel miRNA binding protein QKI which regulated the release of let-7b. Totally speaking, it was an interesting study.
However, central issues of experimental execution and reporting need to be addressed though.

Comments for the author

1.In this MS, the authors only demonstrated that QKI was served as a miRBP that regulated the release of let-7b into exosomes, so I recommend the title of this MS should stress let-7b.
2.The Introduction part of this MS was too brief, the authors should detailed introduce the study background.
3. According to the guideline of MISEV2018(J Extracell Vesicles 2018 Nov 23;7(1):1535750), I recommend the authors use “extracellular vesicles” to replace “exosomes” in the MS. And the authors should identify the characterization of exosomes.
4.The authors should indicate the reason why let- 7b, miR-21, and miR-130b were used to identify miRBPs and also the reason why QKI was chose as a potential miRBP related to miRNA included into exosomes.
5.The authors speculated that the UAA sequence in the YUAAY motif might be the RBD of QKI, the authors should demonstrated it experimentally, such as the RBD mutation experiment.
6.The circulating miRNA was not only carried by exosomes but also combined with RBPs in the circulation, therefore, I recommended to detected the total let-7b expressions in the circulation when QKI was down-regulated.
7.The authors used ultracentrifugation to isolate exsomes, however, this method can not separate exosomes from non-vesicular material.Therefore, I recommend the authors use density gradient centrifugation method to purify exosomes and verified that whether the expressions of let-7b was increased when QKI was down-regulated.
8.The part of Discussion was too brief, the authors should discuss the results profoundly.
9.Language issue: Grammatical errors have also slipped in. Editorial corrections are warranted.

Reviewer 3

Advance summary and potential significance to field

© 2024. Published by The Company of Biologists under the terms of the Creative Commons Attribution License (https://creativecommons.org/licenses/by/4.0/).

In their manuscript Kyung-Won et al. has identified the QKI protein responsible retarded extracellar export of miRNA in mammalian cells. The protein found to be responsible for let-7b retention in the cytoplasm and prevents its entry into exsomal export pathway. The data although are interesting but unfortunately lack some important information. It is an incomplete study and not recommendable for publication in its current form. The major concerns are:
1. The RNA binding capacity of QKI recombinant protein is required to be shown by EMSA assay to show its specificity for miRNA binding. A comment on the Kd of QKI-let-7b RNA complex is required and is needed to be calculated.
2. To rule out that QKI is not restricting miRNA export by indirectly interacting with another RNA binding protein is an important concern. Authors should comment on this possibility. Does this protein interact with other RNA/miRNA binding proteins? i.e. HuR ?
3. The “exosome” population that were measured for export level of miRNA should be characterized by NTA, TEM/AFM and western blot assays for marker proteins. Otherwise it is impossible to conclude that the Exosome analyzed here are free from miRNP and microvesicles which are also used for miRNA export by animal cells.
4. Levels of other members of let-7a family (let-7a and let-7e) should be studied to conclude on the let-7b specific effect of QKI.

Comments for the author

In their manuscript Kyung-Won et al. has identified the QKI protein responsible retarded extracellar export of miRNA in mammalian cells. The protein found to be responsible for let-7b retention in the cytoplasm and prevents its entry into exsomal export pathway. The data although are interesting but unfortunately lack some important information. It is an incomplete study and not recommendable for publication in its current form. The major concerns are:
1. The RNA binding capacity of QKI recombinant protein is required to be shown by EMSA assay to show its specificity for miRNA binding. A comment on the Kd of QKI-let-7b RNA complex is required and is needed to be calculated.
2. To rule out that QKI is not restricting miRNA export by indirectly interacting with another RNA binding protein is an important concern. Authors should comment on this possibility. Does this protein interact with other RNA/miRNA binding proteins? i.e. HuR ?
3. The “exosome” population that were measured for export level of miRNA should be characterized by NTA, TEM/AFM and western blot assays for marker proteins. Otherwise it is impossible to conclude that the Exosome analyzed here are free from miRNP and microvesicles which are also used for miRNA export by animal cells.
4. Levels of other members of let-7a family (let-7a and let-7e) should be studied to conclude on the let-7b specific effect of QKI.

First revision

Author response to reviewers' comments

Responses to the reviewers’ comments

Reviewer 1 Advance Summary and Potential Significance to Field:
This is an interesting, but a descriptive study. The abstract could be rephrased to make it clearer. Discussion must be revised.
[AU] We appreciate the reviewer’s comments. We have rephrased the abstract and revised the discussion as requested.

Reviewer 1 Comments for the Author:
Major Points:
1. Abstract is unclear. State what is the goal of this research and what is new here.
[AU] We appreciate the reviewer’s comments. We revised the abstract clearer and added the goal of this research.

© 2024. Published by The Company of Biologists under the terms of the Creative Commons Attribution License (https://creativecommons.org/licenses/by/4.0/).

2. This is a decsriptive and preliminary study, not mechanistic.

[AU] We appreciate the reviewer's comments. In the revised manuscript, we have included additional data such as affinity of QKI with other miRNAs (miR-21, miR-130b, and N20 random RNA), and mutant let-7b lacking the consensus sequence of QKI binding in **Figure 1D and S1D**. In addition, we have purified EVs by using ultracentrifugation and then examined EVs marker proteins, miRNA abundance, and numbers/sizes of individual EVs in **Figure S4A and 4B**. Thus, we believe that the revised manuscript is improved further with clear molecular mechanisms.

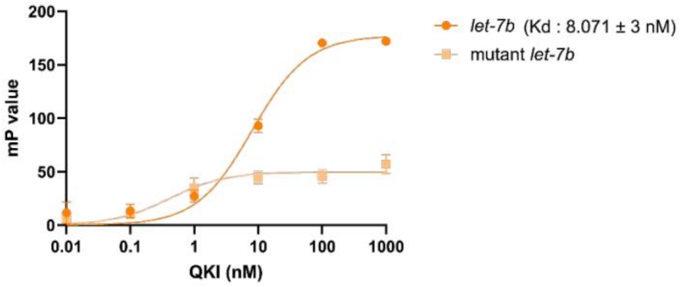

Figure 1D. Anisotropy analysis of recombinant QKI protein with let-7b-Cy3 (5'-rUrGrArGrGrUrArGrUrArGrGrUrUrGrUrGrUrGrGrUrU-Cy3-3, Kd=8.071 ± 3nM) and mutant let- 7b (5'-rUrGrArGrGrUrArGrUrArGrGrUrUrGrCrCrArUrUrArU-Cy3-3').

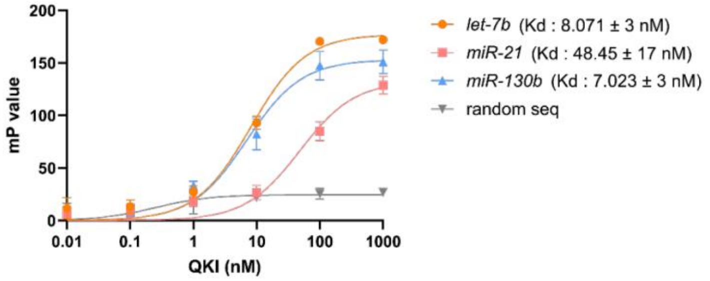

Supplemental Figure S1D. Anisotropy analysis of recombinant QKI protein with let-7b (Kd=8.071±3 nM); miR-21 (Kd=48.45±17 nM); miR-130b (Kd=7.032±3 nM); N20 random RNA (random seq)

A

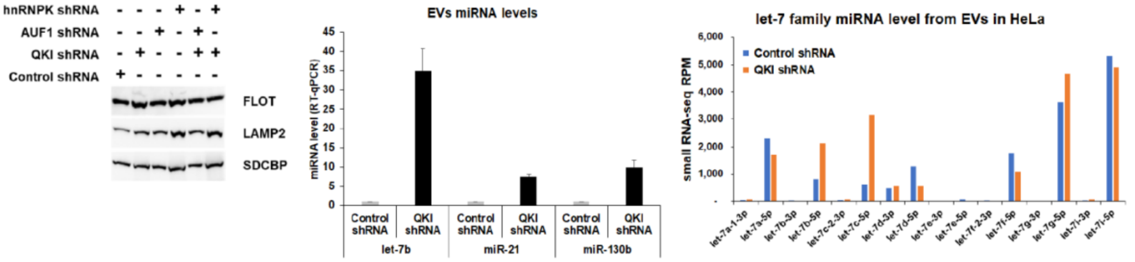

B

Samples	Total number (ea/mL)	Size mean (nm)	Standard Deviation	D10 (nm)	D50 (nm)	D90 (nm)
Control shRNA	5.05 e+11	187.9	69.3	111.6	183.9	293.6
QKI shRNA	2.97 e+11	183.7	83.1	90.1	168.8	266.1
AUF1 shRNA	6.32 e+11	176.1	65.5	110.2	167.4	241.9
hnRNPK shRNA	4.10 e+11	183.7	71	98.2	178.6	264.2
QKI and AUF1 shRNA	4.31 e+11	197.4	84.2	107.3	183.4	315.3
QKI and hnRNPK shRNA	2.31 e+12	178.4	65.8	98.3	174.1	262.5

Supplemental Figure S4. Extracellular Vesicle purification and miRNA quantitation.
(A) Immunoblots of extracellular vesicles marker proteins after transfection of plasmids expressing shRNAs targeting QKI, AUF1, hnRNPK or GFP as a control. The immunoblot images are representative of three independent experiments. RT-qPCR levels of miRNAs purified from the corresponding extracellular vesicles. The graphs are averages and standard deviation of three independent experiments. RNA-seq level (reads per million, RPM) of let-7 family miRNAs purified from extracellular vesicles of QKI or Control shRNA.
(B) Size quantification of extracellular vesicles used in (A).

© 2024. Published by The Company of Biologists under the terms of the Creative Commons Attribution License (https://creativecommons.org/licenses/by/4.0/).

3. Discussion is too short. In the discussion, discuss your results focusing on what is new here, in other words, synthesize new knowledge through discussion.
[AU] We appreciate the reviewer's comments. We have expanded the discussion and emphasized new findings from our current study.

Reviewer 2 Advance Summary and Potential Significance to Field:
In this manuscript, the authors reported a novel miRNA binding protein QKI which regulated the release of let-7b. Totally speaking, it was an interesting study. However, central issues of experimental execution and reporting need to be addressed though.
[AU] We appreciate the reviewer's comments. We have revised the manuscript according to the reviewer's comment and suggestion.

Reviewer 2 Comments for the Author:
1. In this MS, the authors only demonstrated that QKI was served as a miRBP that regulated the release of let-7b into exosomes, so I recommend the title of this MS should stress let-7b.
[AU] We appreciate the reviewer's comments. We revised the title of the manuscript and stresses let- 7b accordingly.

2. The Introduction part of this MS was too brief, the authors should detailed introduce the study background.
[AU] We appreciate the reviewer's comments. We expanded introduction part and detailed the background of this study.

3. According to the guideline of MISEV2018(J Extracell Vesicles 2018 Nov 23;7(1):1535750), I recommend the authors use "extracellular vesicles" to replace "exosomes" in the MS. And the authors should identify the characterization of exosomes.
[AU] We appreciate the reviewer's comments. We have used extracellular vesicles to replace exosomes throughout the revised manuscript.

4. The authors should indicate the reason why let- 7b, miR-21, and miR-130b were used to identify miRBPs and also the reason why QKI was chose as a potential miRBP related to miRNA included into exosomes.
[AU] We appreciate the reviewer's comments. We have used let-7b as a tumor suppressive miRNA, miR-21, an oncogenic miRNA, and miR-130b as an inducible miRNA during differentiation of adipocytes. We have chosen QKI as a miRNA-binding protein based on our previous findings in **Min et al., 2024, *RNA biology***. We have included this explanation in the revised manuscript.

5. The authors speculated that the UAA sequence in the YUAAY motif might be the RBD of QKI, the authors should demonstrated it experimentally, such as the RBD mutation experiment.
[AU] We appreciate the reviewer's comments. We have utilized YUAAY mutant of let-7b and measured dissociation constant with QKI (**Figure 1D**). Our results showed that mutant let-7b failed to bind recombinant QKI as we calculated for mutant let-7b compared to wild type let-7b (Kd=8.071±3 nM). We also measured Kd of miR-21, miR-130b, and N20 random RNA with QKI, and found the Kd values of 48.45±17 nM for miR-21and 7.032±3 nM for miR-130b respectively in **Figure S1D**. Thus, this result has shown that QKI binds let-7b by using its consensus sequence on let-7b. We have included this results in the revised manuscript accordingly.

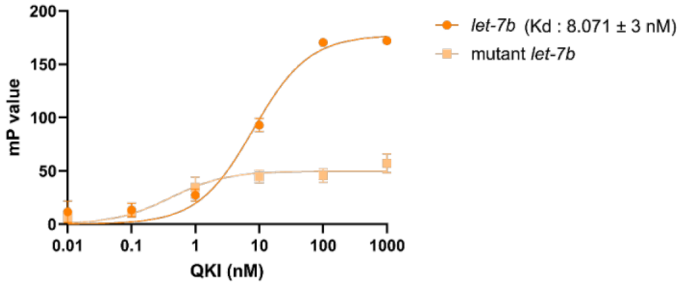

© 2024. Published by The Company of Biologists under the terms of the Creative Commons Attribution License (https://creativecommons.org/licenses/by/4.0/).

Figure 1D. Anisotropy analysis of recombinant QKI protein with let-7b-Cy3 (5'-rUrGrArGrGrUrArGrUrArGrGrUrUrGrUrGrUrGrGrUrU-Cy3-3, Kd=8.071 ± 3nM) and mutant let- 7b (5'-rUrGrArGrGrUrArGrUrArGrGrUrUrGrCrCrArUrUrArU-Cy3-3').

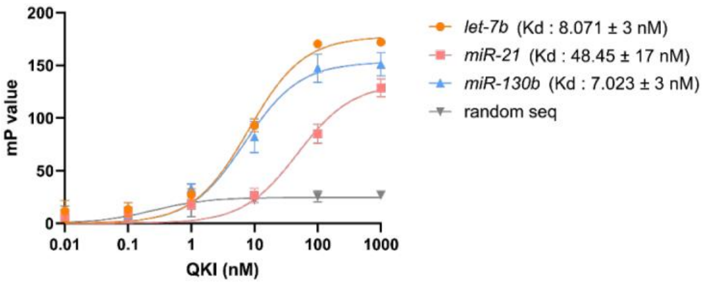

Supplemental Figure S1D. Anisotropy analysis of recombinant QKI protein with let-7b (Kd=8.071±3 nM); miR-21 (Kd=48.45±17 nM); miR-130b (Kd=7.032±3 nM); N20 random RNA (random seq)

6. The circulating miRNA was not only carried by exosomes but also combined with RBPs in the circulation, therefore, I recommended to detected the total let-7b expressions in the circulation when QKI was down-regulated.

[AU] We appreciate the reviewer's comments. We have detected total let-7b expression from whole serum of mice (**Figure on left**) and media from HeLa cell culture (**Figure on right**), and observed a little less change compared to EV let-7b level. These results are included in Figure S4C.

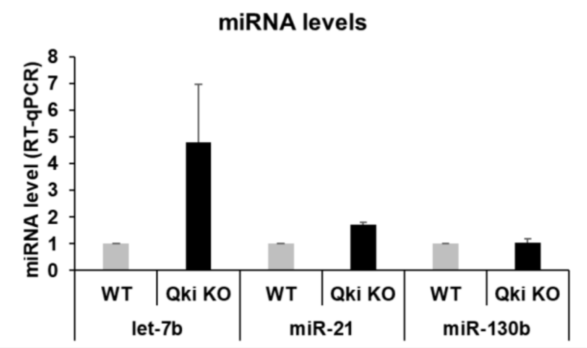

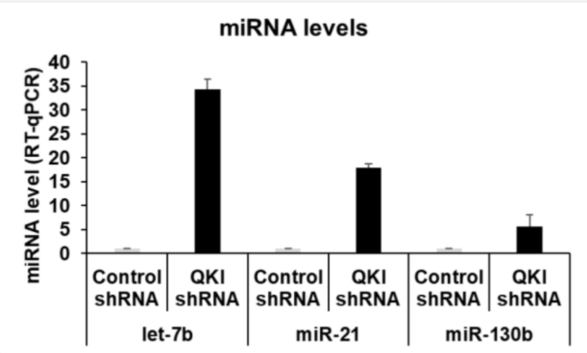

Supplemental Figure 4C. RT-qPCR analysis of let-7b, miR-21, and miR-130b in whole serum from Qki$^{+/+}$ and Qki KO mice, or media from HeLa transfected with QKI or control shRNA plasmid.

7. The authors used ultracentrifugation to isolate exsomes, however, this method can not separate exosomes from non-vesicular material. Therefore, I recommend the authors use density gradient centrifugation method to purify exosomes and verified that whether the expressions of let-7b was increased when QKI was down-regulated.

[AU] We appreciate the reviewer's comments. We have used density gradient centrifugation as performed previously (Kwon et al., 2016 *J Biol Chem*, Lee et al., 2022 *J Extracell Vesicles*, Kang et al., 2024 *J Control Release*), and observed changes of let-7b enrichment comparable to one from the original methods. We have included these results in **the revised Figure S4A and 4B**. Thus, we believe that the revised manuscript is improved further with clear molecular mechanisms.

© 2024. Published by The Company of Biologists under the terms of the Creative Commons Attribution License (https://creativecommons.org/licenses/by/4.0/).

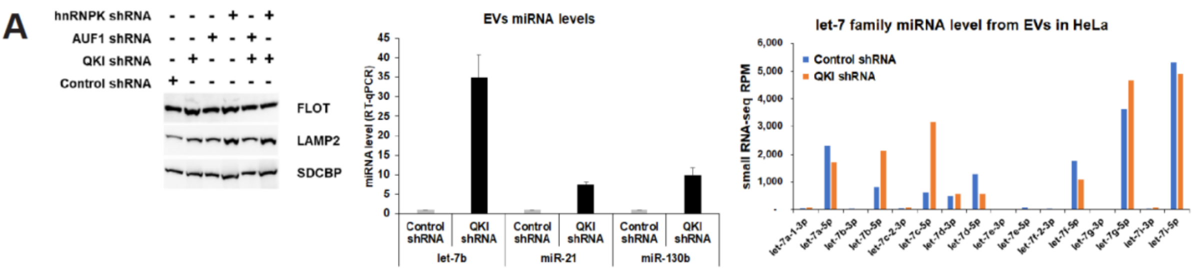

B

Samples	Total number (ea/mL)	Size mean (nm)	Standard Deviation	D10 (nm)	D50 (nm)	D90 (nm)
Control shRNA	5.05 e+11	187.9	69.3	111.6	183.9	293.6
QKI shRNA	2.97 e+11	183.7	83.1	90.1	168.8	266.1
AUF1 shRNA	6.32 e+11	176.1	65.5	110.2	167.4	241.9
hnRNPK shRNA	4.10 e+11	183.7	71	98.2	178.6	264.2
QKI and AUF1 shRNA	4.31 e+11	197.4	84.2	107.3	183.4	315.3
QKI and hnRNPK shRNA	2.31 e+12	178.4	65.8	98.3	174.1	262.5

Supplemental Figure S4. Extracellular Vesicle purification and miRNA quantitation.
(A) Immunoblots of extracellular vesicles marker proteins after transfection of plasmids expressing shRNAs targeting QKI, AUF1, hnRNPK or GFP as a control. The immunoblot images are representative of three independent experiments. RT-qPCR levels of miRNAs purified from the corresponding extracellular vesicles. The graphs are averages and standard deviation of three independent experiments. RNA-seq level (reads per million, RPM) of let-7 family miRNAs purified from extracellular vesicles of QKI or Control shRNA.
(B) Size quantification of extracellular vesicles used in (A).

8. The part of Discussion was too brief, the authors should discuss the results profoundly.

[AU] We appreciate the reviewer’s comments. We have expanded the discussion and included new contents such as the association of QKI with brain diseases and insights into the regulatory mechanism of miRNA loading into extracellular vesicles.

9. Language issue: Grammatical errors have also slipped in. Editorial corrections are warranted.

[AU] We appreciate the reviewer’s comments. We have edited the English grammars with the help of professional editors from Johns Hopkins University.

Reviewer 3 Advance Summary and Potential Significance to Field:

In their manuscript Kyung-Won et al. has identified the QKI protein responsible retarded extracellar export of miRNA in mammalian cells. The protein found to be responsible for let-7b retention in the cytoplasm and prevents its entry into exsomal export pathway. The data although are interesting but unfortunately lack some important information. It is an incomplete study and not recommendable for publication in its current form. The major concerns are:

[AU] We appreciate the reviewer’s comments. We have addressed the reviewer’s concern accordingly.

1. The RNA binding capacity of QKI recombinant protein is required to be shown by EMSA assay to show its specificity for miRNA binding. A comment on the Kd of QKI-let-7b RNA complex is required and is needed to be calculated.

[AU] We appreciate the reviewer’s comments. We have utilized YUAAY mutant of let-7b and measured dissociation constant with QKI (**Figure 1D**). Our results showed that mutant let-7b failed to bind recombinant QKI as we calculated for mutant let-7b compared to wild type let-7b (K_d=8.071±3 nM). We also measured Kd of miR-21, miR-130b, and N20 random RNA with QKI, and found the K_d values of 48.45±17 nM for miR-21and 7.032±3 nM for miR-130b respectively in **Figure S1D**. Thus, this result has shown that QKI binds let-7b by using its consensus sequence on let-7b. We have included this results in the revised manuscript accordingly.

© 2024. Published by The Company of Biologists under the terms of the Creative Commons Attribution License (https://creativecommons.org/licenses/by/4.0/).

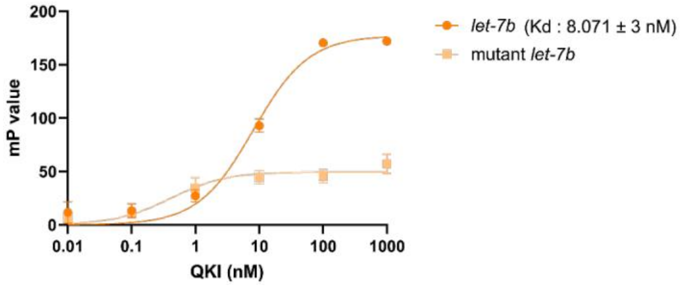

Figure 1D. Anisotropy analysis of recombinant QKI protein with let-7b-Cy3 (5'-rUrGrArGrGrUrArGrUrArGrGrUrUrGrUrGrUrGrGrUrU-Cy3-3, Kd=8.071 ± 3nM) and mutant let- 7b (5'-rUrGrArGrGrUrArGrUrArGrGrUrUrGrCrCrArUrUrArU-Cy3-3').

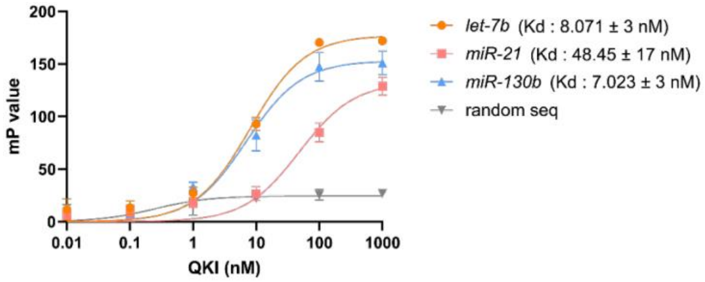

Supplemental Figure S1D. Anisotropy analysis of recombinant QKI protein with let-7b (Kd=8.071±3 nM); miR-21 (Kd=48.45±17 nM); miR-130b (Kd=7.032±3 nM); N20 random RNA (random seq)

2. To rule out that QKI is not restricting miRNA export by indirectly interacting with another RNA binding protein is an important concern. Authors should comment on this possibility. Does this protein interact with other RNA/miRNA binding proteins? i.e. HuR ?

[AU] We appreciate the reviewer's comments. We have commented on this possibility in the discussion of the revised manuscript. In our previous publications, we have found that let-7b also directly binds with other RNA-binding proteins with Kd values of 5.9±0.8 nM for AUF1 p37, 9.7±1.5 nM for AUF1 p42, and 1.4±0.2 nM for HuR respectively (Yoon et al., 2013 *Nature Communications* and 2015 *Genes Dev*).

3. The "exosome" population that were measured for export level of miRNA should be characterized by NTA, TEM/AFM and western blot assays for marker proteins. Otherwise it is impossible to conclude that the Exosome analyzed here are free from miRNP and microvesicles which are also used for miRNA export by animal cells.

[AU] We appreciate the reviewer's comments. We have measured number and sized of exosome, and then included this result in the revised manuscript, **Figure S4B**. We also provided western blots of exosome marker proteins after sucrose density centrifugation as suggested by the reviewer #2 as well (**Figure S4A**). Due to technical difficulty in the current institution, we have not performed TEM/AFM.

© 2024. Published by The Company of Biologists under the terms of the Creative Commons Attribution License (https://creativecommons.org/licenses/by/4.0/).

A

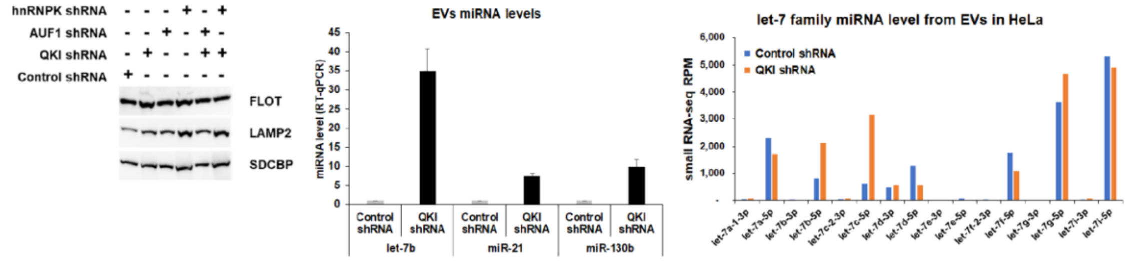

B

Samples	Total number (ea/mL)	Size mean (nm)	Standard Deviation	D10 (nm)	D50 (nm)	D90 (nm)
Control shRNA	5.05 e+11	187.9	69.3	111.6	183.9	293.6
QKI shRNA	2.97 e+11	183.7	83.1	90.1	168.8	266.1
AUF1 shRNA	6.32 e+11	176.1	65.5	110.2	167.4	241.9
hnRNPK shRNA	4.10 e+11	183.7	71	98.2	178.6	264.2
QKI and AUF1 shRNA	4.31 e+11	197.4	84.2	107.3	183.4	315.3
QKI and hnRNPK shRNA	2.31 e+12	178.4	65.8	98.3	174.1	262.5

Supplemental Figure S4. Extracellular Vesicle purification and miRNA quantitation.
(A) Immunoblots of extracellular vesicles marker proteins after transfection of plasmids expressing shRNAs targeting QKI, AUF1, hnRNPK or GFP as a control. The immunoblot images are representative of three independent experiments. RT-qPCR levels of miRNAs purified from the corresponding extracellular vesicles. The graphs are averages and standard deviation of three independent experiments. RNA-seq level (reads per million, RPM) of let-7 family miRNAs purified from extracellular vesicles of QKI or Control shRNA.
(B) Size quantification of extracellular vesicles used in (A).

4. Levels of other members of let-7a family (let-7a and let-7e) should be studied to conclude on the let-7b specific effect of QKI.

[AU] We appreciate the reviewer's comments. We have analyzed abundance of let-7 family miRNAs from small RNA-seq of EVs purified from HeLa and provided this result in **the revised Figure S4A**. Our result demonstrated that QKI suppresses the loading of let-7 family miRNAs, including let-7b- 5p and let-7c-5p into extracellular vesicles.

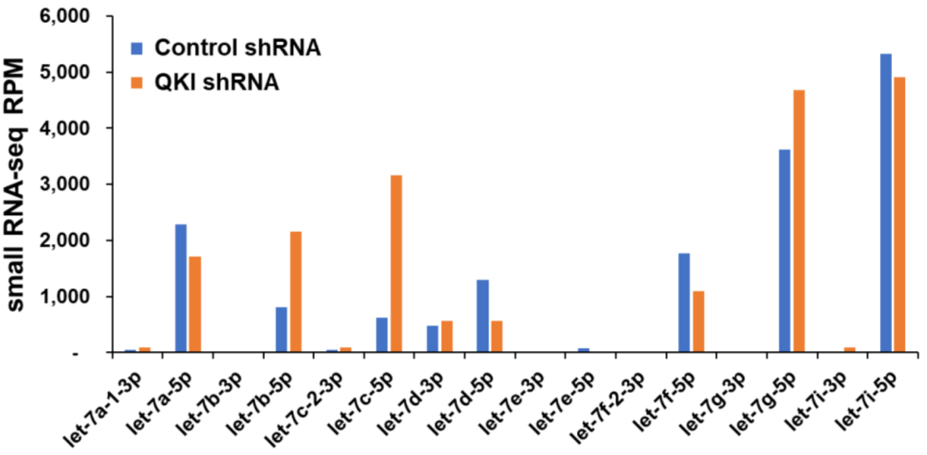

Supplemental Figure S4A. small RNA-seq of let-7 family miRNAs in extracellular vesicles from HeLa cells after transfection of QKI or control shRNA.

Second decision letter

MS ID#: JOCES/2023/261575

MS TITLE: Mature microRNA-binding protein QKI suppresses extracellular microRNA let-7b release

© 2024. Published by The Company of Biologists under the terms of the Creative Commons Attribution License (https://creativecommons.org/licenses/by/4.0/).

AUTHORS: Kyung-Won Min, Kyoung-Min Choi, Hyejin Mun, Seungbeom Ko, Ji Won Lee, Cari A Sagum, Mark T Bedford, Young-Kook Kim, Joe R Delaney, Jung-Hyun Cho, Ted M Dawson, Valina L Dawson, Waleed Twal, Dong-Chan Kim, Clarisse H Panganiban, Hainan Lang, Xin Zhou, Seul A Shin, Jian Hu, Tilman Heise, Sang-Ho Kwon, Dongsan Kim, Young Hwa Kim, Sung-Ung Kang, Kyungmin Kim, Sydney Lewis, Ahmet Eroglu, Seonghyun Ryu, Dongin Kim, Jeong Ho Chang, Junyang Jung, and Je-Hyun Yoon

We have now reached a decision on the above manuscript.

To see the reviewers' reports and a copy of this decision letter, please go to: https://submit-jcs.biologists.org and click on the 'Manuscripts with Decisions' queue in the Author Area. (Corresponding author only has access to reviews.)

As you will see, the reviewers gave favourable reports but suggested some further improvements to the discussion in your revised manuscript. I hope that you will be able to carry these out because I would like to be able to accept your paper soon.

Please ensure that you clearly highlight all changes made in the revised manuscript. Please avoid using 'Tracked changes' in Word files as these are lost in PDF conversion.

I should be grateful if you would also provide a point-by-point response detailing how you have dealt with the points raised by the reviewers in the 'Response to Reviewers' box. Please attend to all of the reviewers' comments. If you do not agree with any of their criticisms or suggestions please explain clearly why this is so.

Reviewer 1

Advance summary and potential significance to field

The revised manuscript is improved with new data and revised abstract; but the discussion did not adequately synthesize new knowledge. What is the novelty? why should the readers care?

Comments for the author

The authors mostly addressed all of my concerns, however, the discussion could have been improved further. I wonder if the corresponding/senior author read and revised this version.

Reviewer 2

Advance summary and potential significance to field

In this manuscript, the authors reported a novel miRNA binding protein QKI which regulated the release of let-7b. Totally speaking, it was an interesting study.

Comments for the author

I suggeseted to publish this manuscript in the JCS.

Reviewer 3

Advance summary and potential significance to field

The authors have addressed majority of my concern experimentally or by providing appropriate discussion. I have no major issue left with the revised version. However, the authors new data suggest the effect of KI on other members of the let-7a family. The significance in the context of

© 2024. Published by The Company of Biologists under the terms of the Creative Commons Attribution License (https://creativecommons.org/licenses/by/4.0/).

inflammation should be discussed and the title should be changed as it is not exclusive effect on let-7b only.

Comments for the author

The authors have addressed majority of my concern experimentally or by providing appropriate discussion. I have no major issue left with the revised version. However, the authors new data suggest the effect of KI on other members of the let-7a family. The significance in the context of inflammation should be discussed and the title should be changed as it is not exclusive effect on let-7b only.

Second revision

Author response to reviewers' comments

Responses to the reviewers’ comments

Reviewer 1 Advance Summary and Potential Significance to Field:
The revised manuscript is improved with new data and revised abstract; but the discussion did not adequately synthesize new knowledge. What is the novelty? why should the readers care?
[AU] We appreciate the reviewer’s comments. In the revised manuscript, we emphasized the possibility that QKI plays a key role in modulating the function of mature let-7b and acts as a master regulator of brain inflammation.

Reviewer 1 Comments for the Author:
The authors mostly addressed all of my concerns, however, the discussion could have been improved further. I wonder if the corresponding/senior author read and revised this version.
[AU] We appreciate the reviewer’s comments. We, especially the senior author, have revised the discussion to highlight the novel aspects of our research according to the reviewer’s comment and suggestion.

Reviewer 2 Advance Summary and Potential Significance to Field:
In this manuscript, the authors reported a novel miRNA binding protein QKI which regulated the release of let-7b. Totally speaking, it was an interesting study.
[AU] We appreciate the reviewer’s comments.

Reviewer 2 Comments for the Author:
I suggeseted to publish this manuscript in the JCS.
[AU] We appreciate the reviewer’s comments.

Reviewer 3 Advance Summary and Potential Significance to Field:
The authors have addressed majority of my concern experimentally or by providing appropriate discussion. I have no major issue left with the revised version. However, the authors new data suggest the effect of KI on other members of the let-7a family. The significance in the context of inflammation should be discussed and the title should be changed as it is not exclusive effect on let- 7b only.
[AU] We appreciate the reviewer’s comments. We discussed the effect of let-7c loading into extracellular vesicles due to QKI depletion on inflammation, according to the reviewer’s comments and suggestions. Although we showed that let-7b and let-7c were enriched in extracellular vesicles after silencing QKI in HeLa cells (**Figure S4A**), there will be low chance that let-7c could act as a ligand of TLR7. Therefore, we prefer to keep the original title, even though we agree with the reviewer’s suggestion.

Third decision letter

MS ID#: JOCES/2023/261575

© 2024. Published by The Company of Biologists under the terms of the Creative Commons Attribution License (https://creativecommons.org/licenses/by/4.0/).

MS TITLE: Mature microRNA-binding protein QKI suppresses extracellular microRNA let-7b release

AUTHORS: Kyung-Won Min, Kyoung-Min Choi, Hyejin Mun, Seungbeom Ko, Ji Won Lee, Cari A Sagum, Mark T Bedford, Young-Kook Kim, Joe R Delaney, Jung-Hyun Cho, Ted M Dawson, Valina L Dawson, Waleed Twal, Dong-Chan Kim, Clarisse H Panganiban, Hainan Lang, Xin Zhou, Seul A Shin, Jian Hu, Tilman Heise, Sang-Ho Kwon, Dongsan Kim, Young Hwa Kim, Sung-Ung Kang, Kyungmin Kim, Sydney Lewis, Ahmet Eroglu, Seonghyun Ryu, Dongin Kim, Jeong Ho Chang, Junyang Jung, and Je-Hyun Yoon

I am happy to tell you that your manuscript has been accepted for publication in Journal of Cell Science, pending standard publication integrity checks.

© 2024. Published by The Company of Biologists under the terms of the Creative Commons Attribution License (https://creativecommons.org/licenses/by/4.0/).
